# KRAS: the Achilles’ heel of pancreas cancer biology

**DOI:** 10.1172/JCI191939

**Published:** 2025-08-15

**Authors:** Kristina Drizyte-Miller, Taiwo Talabi, Ashwin Somasundaram, Adrienne D. Cox, Channing J. Der

**Affiliations:** 1 Lineberger Comprehensive Cancer Center,; 2 Department of Medicine,; 3 Department of Pharmacology, and; 4 Department of Radiation Oncology, University of North Carolina at Chapel Hill, Chapel Hill, North Carolina, USA.

## Abstract

The genetic landscape of pancreatic ductal adenocarcinoma (PDAC) is well-established and dominated by four key genetic driver mutations. Mutational activation of the *KRAS* oncogene is the initiating genetic event, followed by genetic loss of function of the *CDKN2A*, *TP53*, and *SMAD4* tumor suppressor genes. Disappointingly, this information has not been leveraged to develop clinically effective targeted therapies for PDAC treatment, where current standards of care remain cocktails of conventional cytotoxic drugs. Nearly all (~95%) PDAC harbors *KRAS* mutations, and experimental studies have validated the essential role of *KRAS* mutation in PDAC tumorigenic and metastatic growth. Identified in 1982 as the first gene shown to be aberrantly activated in human cancer, *KRAS* has been the focus of intensive drug discovery efforts. Widely considered “undruggable,” KRAS has been the elephant in the room for PDAC treatment. This perception was shattered recently with the approval of two KRAS inhibitors for the treatment of *KRAS^G12C^*-mutant lung and colorectal cancer, fueling hope that KRAS inhibitors will lead to a breakthrough in PDAC therapy. In this Review, we summarize the key role of aberrant KRAS signaling in the biology of pancreatic cancer; provide an overview of past, current, and emerging anti-KRAS treatment strategies; and discuss current challenges that limit the clinical efficacy of directly targeting KRAS for pancreatic cancer treatment.

## Introduction

Following lung and colorectal cancer (CRC), pancreatic cancer is the third leading cause of cancer deaths in the United States (1). Whereas recent decades have seen declines in the mortality rates for lung cancer and CRC, pancreatic cancer mortality rates have gradually increased, in part because of the obesity epidemic. Indeed, pancreatic cancer is projected to surpass CRC and become the second leading cause of cancer-related mortality by 2040 (2). Although its 5-year overall survival (OS) rate has improved from 4% in the mid-1990s to 13%, it remains among the lowest of all cancer types (1).

Pancreatic ductal adenocarcinoma (PDAC), an exocrine neoplasm, is the most common subtype of pancreatic cancer, accounting for over 90% of pancreatic neoplasms (3). Despite a well-defined genetic landscape of PDAC (4), no effective targeted therapies have been approved for the majority of patients with PDAC, and the standard of care remains surgery and chemotherapy (5). Most patients are diagnosed with advanced metastatic disease (1), with only 15%–20% eligible for surgery at diagnosis (6). For unresectable PDAC, the first-line therapy is either a combination of 5-fluorouracil, leucovorin, irinotecan, and oxaliplatin, termed FOLFIRINOX (7), or the combination of gemcitabine and nanoparticle albumin-bound paclitaxel (nab-paclitaxel) (8). Disappointingly, the current standard of care is associated with high toxicity, and the median OS on a first-line therapy is less than 12 months and even lower (less than 7 months) on a second-line therapy (7, 8).

The Kirsten rat sarcoma (*KRAS*) oncogene was identified originally as a retroviral gene responsible for the oncogenic properties of the Kirsten murine sarcoma virus and was later determined to have been transduced from the normal rat genome (Table 1). The discovery of activated KRAS oncogenes in human cancer cell lines in 1982 (9, 10), and their establishment as a sufficient (11) and necessary driver of PDAC growth (12–14), supported the potential significance of KRAS-targeted therapies for PDAC treatment. However, KRAS was initially considered an undruggable cancer target (15). Early efforts focused on indirect strategies to inhibit KRAS membrane association and downstream effector signaling but with minimal therapeutic success (16). It took nearly 40 years until the first direct KRAS inhibitors, targeting a specific mutation (KRAS^G12C^), were clinically approved for non–small cell lung cancer (NSCLC) treatment (17, 18). The successful development of direct KRAS^G12C^ inhibitors had a tsunami effect on drug discovery, with more than 50 mutation-selective and pan/multi KRAS/RAS inhibitors now under clinical evaluation (16, 19) (Supplemental Table 1; supplemental material available online with this article; https://doi.org/10.1172/JCI191939DS1).

In this Review, we focus on KRAS as the Achilles’ heel of pancreatic cancer treatment. It is both the critical driver of PDAC growth as well as arguably the greatest therapeutic vulnerability for PDAC treatment. We revisit the early indirect strategies of targeting KRAS and provide an overview of the current landscape of direct KRAS inhibitors. We end with a discussion of lessons learned from the results from ongoing clinical trials, resistance mechanisms to KRAS inhibitors, and potential combination strategies to improve outcomes for patients with pancreatic cancer.

## KRAS — the driver of pancreatic cancer

**Figure 1:**
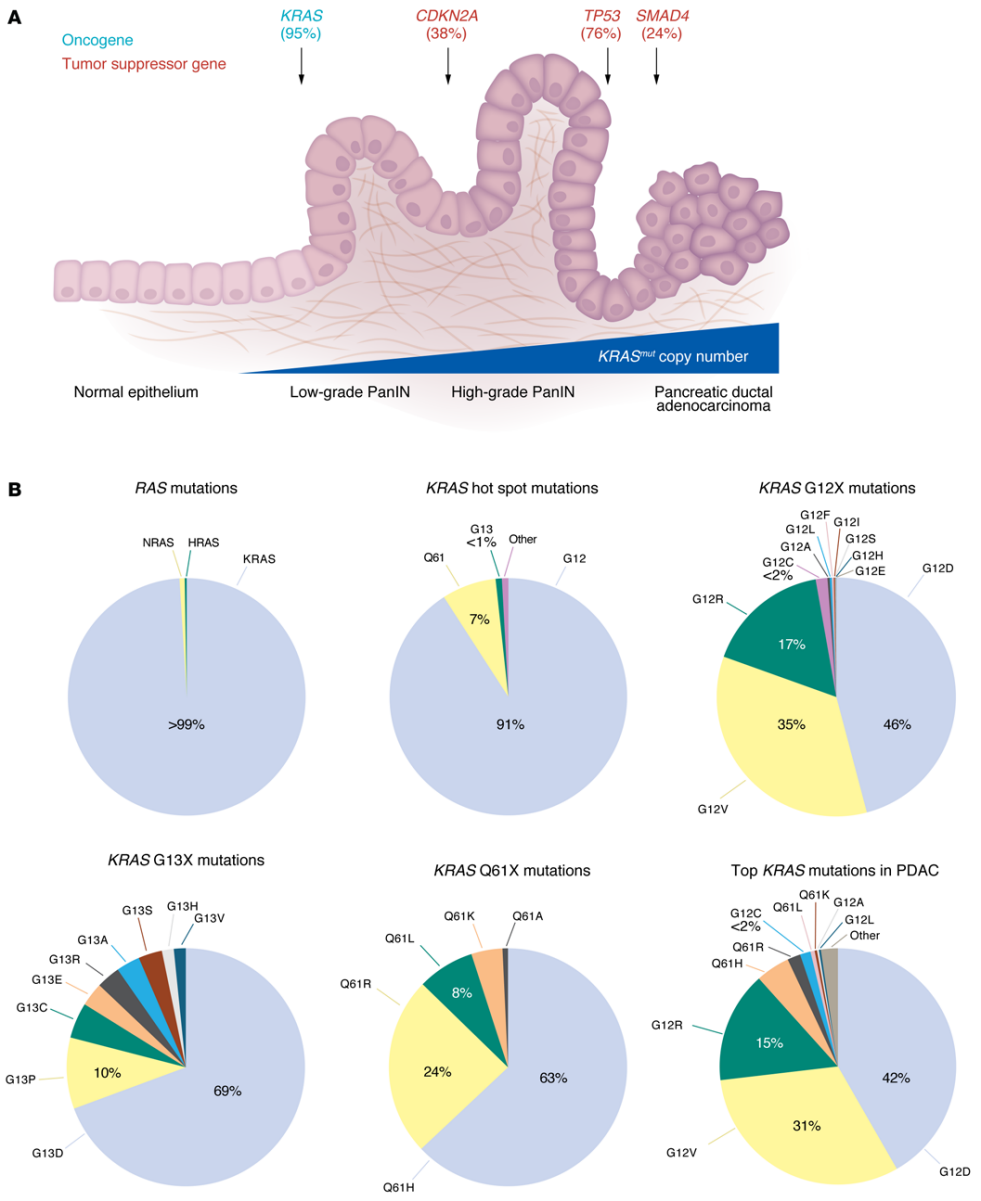
*KRAS* mutations in PDAC. (**A**) Schematic illustrating pancreatic ductal adenocarcinoma (PDAC) pathogenesis and progression (adapted from ref. 176 with permission from Springer Nature Limited, which retains the rights to the reference image). Mutations in *KRAS* oncogene are the initiating step in PDAC development, and they induce transformation of normal pancreas epithelium to low-grade pancreatic intraepithelial neoplasia (PanIN). Progression from low-grade PanINs to high-grade PanINs and eventually invasive PDAC is caused by loss-of-function mutations in *CDKN2A*, *TP53*, and *SMAD4* tumor suppressor genes. The severity of disease is also associated with increased *KRAS^mut^* copy numbers. (**B**) *KRAS* mutation frequencies in PDAC. Data were compiled from the cBioPortal GENIE Cohort v17.0 database (48) from 7,407 patients with PDAC. Of the three *RAS* isoforms, *KRAS* is the predominantly mutated isoform, with *NRAS* and *HRAS* mutations accounting for <1% of PDAC cases. Of the three mutational hot spots, G12X mutations are most common in PDAC, with G12D, G12V, and G12R representing the predominant amino acid mutations at this position. G13X mutations are rare in PDAC and comprise less than 1% of *KRAS* mutations. Q61X mutations are also uncommon, accounting for 7% of *KRAS* point mutations, with Q61H representing the predominant mutation. The authors would like to acknowledge the American Association for Cancer Research and its financial and material support in the development of the AACR Project GENIE registry, as well as members of the consortium for their commitment to data sharing. Interpretations are the responsibility of the authors.

Molecular and histological profiling demonstrated that approximately 85%–90% of PDAC is initiated from the precursor lesions, termed pancreatic intraepithelial neoplasms (PanINs) (Figure 1A), with the remaining 10%–15% arising from mucinous pancreatic cyst precursors, most often intraductal papillary mucinous neoplasms (IPMNs) (20, 21). These precursor lesions undergo a stepwise accumulation of gain- and loss-of-function genetic mutations as they progress to invasive and metastatic PDAC (22).

Genome-wide sequence analyses have identified four predominant gene mutations in PDAC (23–31). In addition to gain-of-function *KRAS* missense mutations, loss-of-function mutations in *CDKN2A* (with additional loss mediated by homozygous deletion or promoter hypermethylation), *TP53*, and/or *SMAD4* tumor suppressor genes dominate the genetic landscape of PDAC (Figure 1A). Genetic profiling of early-stage preneoplastic lesions supports a model in which mutations in these genes contribute to the initiation of neoplasia and progression to invasive and metastatic PDAC (4). While the cell of origin of PDAC is still debated, genetic studies in mouse models support development of an acinar-to-ductal metaplasia in the epithelia of the exocrine pancreas upon acquisition of oncogenic mutations in *Kras* (32). Recent clinicogenomic profiling of 2,336 tumors from patients with both resectable and metastatic PDAC found *KRAS* mutations in 95% of cases, followed by *TP53* mutation in 76%, *CDKN2A/B* mutation in 38%, and *SMAD4* mutation in 24% of cases (33).

Genetically engineered mouse models (GEMMs) have been instrumental tools in establishing *KRAS* mutations as the initiating event in PDAC tumorigenesis (34, 35) (Table 1). Conditional expression of *Kras^G12D^* in pancreatic progenitor cells was sufficient to induce the formation of PanIN lesions that histologically recapitulated the PanIN stages observed in human PDAC, characterized by long latencies and low penetrance of invasive and metastatic disease (11). However, when mutant *Kras* was combined with inactivation of the *Trp53* (36), *Ink4a/Arf* (37), or *Smad4* (38) tumor suppressor genes, it resulted in rapid progression of PanINs and fully penetrant development of invasive and highly metastatic PDAC.

The role of *KRAS* mutations as the critical initiating genetic step in PDAC is also supported by genetic profiling of PanIN lesions, which are characterized as low-grade (LG) and high-grade (HG) PanINs (39) (Figure 1A). *KRAS* but not tumor suppressor mutations are found in LG PanINs (40, 41). Deletions and mutations in *CDKN2A* (encoding p16INK4a and p14ARF) are found in LG PanINs and increase in frequency in HG PanINs, with additional loss of *CDKN2A* expression mediated by promoter hypermethylation (42). *TP53* mutations are found in HG PanINs and increase in frequency in PDAC, whereas *SMAD4* mutations are found in advanced PDAC. *KRAS* mutations are already present in 95% of LG and HG PanIN lesions, consistent with their initiating role (40, 41, 43). However, LG and HG PanIN lesions are present in cancer-free elderly individuals, indicating that additional genetic steps are essential to unleash the oncogenic driver function of mutant KRAS (44).

GEMMs have been key in establishing that mutant *KRAS* is also necessary for tumor maintenance (Table 1). Specifically, inactivation of mutant *Kras^G12D^* in early-stage or established PanINs caused regression of primary (12, 14, 16) and metastatic (13) tumors. Similarly, genetic silencing by RNA interference (RNAi) in *KRAS*-mutant human cancer cell lines also caused growth suppression (45, 46). Together, these findings support targeting KRAS as a therapeutic approach in advanced PDAC.

## KRAS mutations

Approximately 20% of all human cancers harbor *RAS* mutations (47); *KRAS* is the most frequently mutated isoform (83%), followed by *NRAS* (15%), with *HRAS* mutated infrequently (2%) (GENIE Cohort v17.0; ref. 48). *RAS* mutations are seen predominantly at one of three mutational hot spots: glycine-12 (G12), glycine-13 (G13), and glutamine-61 (Q61). The frequency of specific *RAS* gene mutations is highly skewed, with the majority of cancers predominantly expressing a mutation of one specific *RAS* allele (49–51). PDAC is characterized by mutations near-exclusively in *KRAS* (99%), with *HRAS* and *NRAS* mutations occurring in 0.7% and 0.3% of patients with PDAC, respectively (GENIE Cohort v17.0; ref. 48) (Figure 1B). Most missense mutations in *KRAS* occur at the G12 (91%) and Q61 (7%) amino acid positions, with G13 mutations being rare (1%). G12D is the most frequent substitution (41%), followed by G12V (32%) and G12R (16%). This mutation profile is nearly identical to that seen in PanIN lesions (43, 52), further supporting *KRAS* mutation as the initiating genetic event in PDAC.

The prominence of G12R mutations in PDAC contrasts strikingly with other cancers that harbor high levels of *KRAS* mutations; G12R mutations are found in only 1%–2% of NSCLC and CRC (16). Conversely, the smoking-associated *KRAS^G12C^* mutation is the most prevalent *KRAS* mutation in NSCLC (40%), but it is found in less than 2% of PDAC (53). G13X mutations comprise 18% of *KRAS* mutations in CRC, yet they represent less than 1% of *KRAS* mutations in PDAC. The basis for mutation of a specific *RAS* gene or for mutation at specific hot spots in different cancer types remains poorly understood (54). There is evidence for both DNA mutagenic mechanisms as well as biological properties as influencing these frequencies (55, 56). This topic has been addressed in considerable detail in other recent reviews (50, 54, 57).

An emerging concept is that different mutations have distinct consequences for KRAS oncogenic function and, consequently, may exhibit differential therapeutic vulnerabilities (57). Evaluation of patients with PDAC indicates that the different *KRAS* mutations are associated with different clinical characteristics. *KRAS^G12D^* has been associated with the worst survival, whereas *KRAS^G12R^* has been associated with improved survival (33, 52). Compared with *KRAS^G12D^*, *KRAS^G12R^* mutant PDAC also has a less invasive phenotype, enriched in early-stage (stage I) versus late-stage (stage II–III) disease (44% versus 24%), and diminished metastatic potential, with increased lymph node negativity (47% versus 26%). These clinical differences suggest that *KRAS^G12R^* is a less potent cancer driver and are consistent with preclinical GEMM analyses where *Kras^G12R^* did not effectively drive PanIN formation (58). A possible mechanistic basis for the reduced oncogenic potency of *KRAS*^G12R^ may be based in part on reduced migration potential (52) as well as impaired phosphoinoside-3-kinase (PI3K) effector activation and promotion of macropinocytosis (59), a metabolic activity essential for PDAC tumorigenicity (60).

Gene dosage has also emerged as an important parameter that may support mutant *KRAS* driver function and impact the clinical disease and responses to therapy (33). Increased copy numbers of mutant versus WT *KRAS* alleles were associated with worse OS in both resectable (23 months versus 32 months) and metastatic (8.5 months versus 13 months) disease. Mechanisms that increase *KRAS* mutant copy number include preferential amplification of the mutant allele and loss of the WT allele. Whole-genome duplication, which is seen in nearly two-thirds of patients (25), also enhances mutant allele copy numbers. *KRAS* gene amplification is also associated with acquired resistance to direct KRAS inhibitors (16). Finally, GEMM studies support a potential tumor suppressor function of the *KRAS* WT allele (61–63). Consistent with this, loss of the WT allele in patients with *KRAS* mutant copy gains has been associated with significantly worse OS (33). These observations should be taken into consideration when prioritizing the development of therapeutic approaches for patients harboring a particular *KRAS* allele mutation or amplification. The development of highly potent inhibitors against the three most prevalent *KRAS* mutations in PDAC (G12D, G12V, and G12R), along with agents that would sustain pathway inhibition in *KRAS-*amplified tumors, might yield the most impactful therapeutic benefit for patients with PDAC.

Among the 5% of PDAC that are *KRAS* WT, 60% exhibit genetic alterations in the upstream receptor tyrosine kinases (RTKs) (e.g., *NTRK1, NTRK3, FGFR2, ERBB2, ROS1,* and *MET*), at the level of RAS or RAS regulation (e.g., *NRAS, NF1*) or in components of the downstream RAF/MEK/ERK MAPK cascade (e.g., *BRAF, RAF1* and *MAP2K1* (which encodes MEK1) (33). The remaining 40% of *KRAS* WT PDAC lack mutations in the ERK MAPK signaling network and instead are enriched in *GNAS*, *SMARCB1*, and *PIK3CA* mutations. *KRAS* WT PDAC, with and without other ERK MAPK network mutations, exhibits improved OS and response to therapy (33, 52).

## KRAS signaling in PDAC

**Figure 2:**
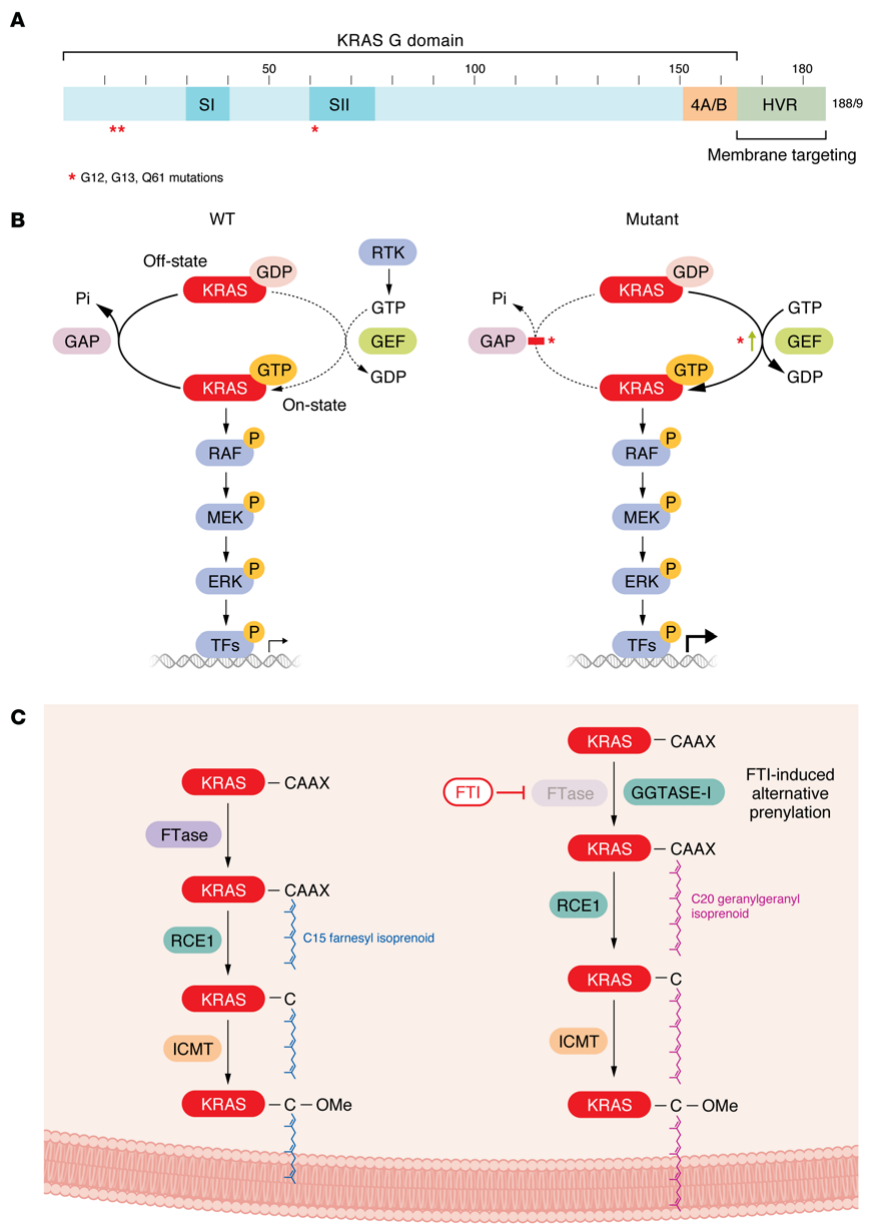
KRAS GTPase regulation and signaling. (**A**) *KRAS* encodes a small GTPase comprising the G domain and hypervariable region (HVR). Alternative splicing of exon four results in two KRAS isoforms (KRAS4A/KRAS4B, denoted as 4A/B), which differ in their carboxyl-terminal 151–188/189 amino acids. The G domain is involved in guanosine triphosphate (GTP) and guanosine diphosphate (GDP) binding and interactions with guanine nucleotide exchange factors (GEFs), GTPase-activating proteins (GAPs), and effectors. HVR contains the CAAX tetrapeptide motif that acts as a signal for posttranslational modifications that promote KRAS plasma membrane association essential for KRAS oncogenic function. Switch I and II regions (denoted as SI and SII) are highlighted, and mutational hot spots at G12, G13, and Q61 positions are indicated with red asterisks. (**B**) KRAS cycles between active GTP-bound and inactive GDP-bound states. Receptor tyrosine kinase (RTK) signaling promotes GEF-mediated GTP loading and activation of KRAS, which then engages downstream effector signaling (i.e., the RAF/MEK/ERK MAPK cascade). GAPs accelerate intrinsic KRAS GTPase activity and GTP hydrolysis to return KRAS to the inactive GDP-bound state. Amino acid substitutions at G12, G13, and Q61 hot spot positions accelerate GDP to GTP exchange rates and/or impair intrinsic or GAP-induced GTP hydrolysis, resulting in constitutively active KRAS. (**C**) KRAS undergoes three posttranslational modifications at the carboxyl-terminal CAAX motif (where C denotes cysteine, A denotes aliphatic, and X denotes terminal residues), which is required for association with membranes. Farnesyltransferase (FTase) adds a 15-carbon farnesyl group to the cysteine amino acid at the CAAX motif, RAS-converting enzyme (RCE1) removes -AAX residues, and isoprenylcysteine carboxylmethyltransferase (ICMT) catalyzes carboxylmethylation of farnesylated cysteine. Inhibition of FTase (FTIs) leads to alternative prenylation of KRAS by geranylgeranyltransferase-I (GGTase-I), which adds a 20-carbon geranylgeranyl group and facilitates KRAS associate with membranes. C, cysteine; Ome, carboxyl methylation.

*KRAS* encodes two highly similar (~85% amino acid identity) isoforms (KRAS4A and KRAS4B) that are produced by alternative splicing of exon four and differ solely in their carboxyl-terminal residues (64) (Figure 2A). KRAS is a small GTPase that functions as a binary on-off switch that relays extracellular signal-induced stimuli to cytoplasmic signaling networks. It comprises an amino-terminal catalytic G domain responsible for binding and hydrolyzing GTP to GDP and a carboxyl-terminal hypervariable region (HVR), which undergoes posttranslational lipid modifications critical for membrane targeting (65–68).

The intrinsically low GTP hydrolysis and exchange activities of WT KRAS are accelerated by guanine nucleotide exchange factors (GEFs) and GTPase-activating proteins (GAPs), respectively (69, 70) (Figure 2B). GEFs (e.g., SOS1) assist in GTP/GDP exchange while GAPs (e.g., NF1) facilitate hydrolysis of the bound GTP. Cycling between the GTP-bound on-state (ON) and GDP-bound off-state (OFF) causes conformational changes in the switch I (residues 30–40) and switch II (residues 60–76) regions of KRAS that are responsible for effector binding and interaction with GEFs and GAPs, respectively (68). The mutational hot spots in *KRAS* occur near the switch regions and, to varying degrees, reduce both intrinsic and GAP-induced GTP hydrolysis and/or increase intrinsic GDP/GTP exchange rates, both of which favor formation of the constitutively ON KRAS (71).

Canonically, KRAS is activated in response to extracellular stimuli to promote cell growth and survival (Figure 2B). Growth factor–mediated RTK (e.g., EGFR) signaling leads to activation of KRAS and subsequent association with downstream effectors to initiate a multitude of signaling pathways. Although more than 12 functional classes comprising >50 validated/putative RAS effectors have been identified (72, 73), the RAF/MEK/ERK MAPK and the PI3K/AKT/mTOR signaling networks comprise the two best validated effector signaling networks that support KRAS-driven oncogenesis (16).

GTP-bound KRAS, whether WT or mutant, promotes activation of RAF serine/threonine kinases (ARAF, BRAF, and RAF1/CRAF) by a complex mechanism involving relief of autoinhibition, promotion of membrane association, phosphorylation by membrane-associated protein kinases, and dimerization. Activated RAF then phosphorylates and activates the MEK1/2 dual-specificity protein kinases, which then phosphorylate and activate the ERK1/2 serine/threonine kinases (74, 75). In contrast to the limited substrates of RAF and MEK, activated ERK regulates a complex and dynamic phosphoproteome in *KRAS*-mutant PDAC cells comprising over 2,000 cytoplasmic and nuclear proteins (76). ERK substrates include transcription factors, kinases, epigenetic regulators, E3 ligases, and phosphatases, which in turn alter gene transcription and cellular signaling to promote cell cycle progression and cell growth (77). One of the key downstream substrates of ERK is the transcription factor MYC (76, 77), which regulates tumor metabolism (78) and is critical for *KRAS*-mutant PDAC growth (79–81).

A second major effector of KRAS is PI3K, which converts phosphatidylinositol-4,5-bisphosphate (PIP2) to phosphatidylinositol-3,4,5-triphosphate (PIP3) to activate the AKT1-3 serine/threonine protein kinases (82). Activated AKT then leads to activation of the kinase mTOR and regulation of cell metabolism, proliferation, migration, and survival (83). PI3K signaling has been shown to be essential for KRAS-driven tumorigenesis in vivo, where mutations in *Pik3ca* (encoding the p110α subunit of PI3K) that result in defective binding to RAS also prevent Kras-driven NSCLC formation and maintenance (84, 85).

Several key findings suggest that the RAF/MEK/ERK MAPK signaling cascade is the major effector of *KRAS*-driven progression and growth of PDAC. First, activating mutations in *Braf*, but not *Pik3ca*, in mice phenocopied *Kras* mutations in driving PDAC initiation and maintenance when coupled with loss of *Tp53* (86). Second, the KRAS-dependent transcriptome and phosphoproteome were nearly identical to the ERK-regulated transcriptome and phosphoproteome in PDAC cells (76, 77). Third, constitutive activation of MEK1 and ERK1/2, but not AKT, rescued KRAS inhibitor-induced growth suppression in PDAC (77). However, mutations in components of the PI3K/AKT/mTOR pathway have been identified in patients who relapsed on KRAS^G12C^ inhibitor treatment (87, 88), suggesting that the role of PI3K and other KRAS effectors in *KRAS*-mutant PDAC needs to be further investigated.

## Early approaches for anti-KRAS therapies in PDAC

### Farnesyltransferase inhibitors.

Initial attempts to directly drug KRAS by developing GTP analogs that would compete with GTP binding or GAP-like molecules that would restore the intrinsic GTPase activity were not successful. Instead, the focus shifted toward targeting the farnesyltransferase (FTase) enzyme responsible for adding a 15-carbon farnesyl lipid modification to the carboxyl-terminus of KRAS (Figure 2C). This modification was shown to be required for KRAS association with plasma membrane and for downstream signaling and cell transformation (49). However, FTase inhibitors (FTIs) had disappointing clinical outcomes with no significant efficacy in *KRAS*-mutant PDAC (89–91) (Table 2). In retrospect, these negative outcomes were predicted by earlier experimental studies that found that FTIs were effective against HRAS- but not KRAS-transformed rodent fibroblasts (92). The explanation for this distinction was that, when FTase activity is blocked, KRAS but not HRAS undergoes alternative prenylation by the FTase-related enzyme, geranylgeranyltransferase-I (GGTase-I), which adds a 20-carbon geranylgeranyl lipid modification to the carboxyl-terminus of KRAS (93, 94) (Figure 2C). This unexpected property of KRAS (and NRAS) was missed by initial studies that focused on *HRAS*-mutant cell models (92, 95, 96), when it was widely believed that the three RAS proteins were identical in biochemical properties and function. Although therapeutic strategies focused on inhibiting KRAS membrane association are still being pursued, these indirect strategies will likely be limited by additional effects on the functions of non-RAS targets.

### Targeting KRAS effector signaling.

With over 100 approved oncology drugs, protein kinases are among the most successful class of anticancer targets (97, 98). Thus, the discovery that the ERK/MAPK cascade is a key effector of KRAS-driven cancer growth fueled a second major approach of indirectly targeting KRAS (Table 2). Multiple small-molecule inhibitors of each node of the RAF/MEK/ERK MAPK cascade have been developed and have shown promise in preclinical studies (99, 100). RAF and MEK inhibitors have been approved for *BRAF*-mutant melanoma and other cancers (101), and one MEK inhibitor has been approved for *NF1*-deficient plexiform neurofibromas (102). However, the use of ERK MAPK inhibitors for the treatment of PDAC and other *KRAS*-mutant cancers has been challenging owing to on-target toxicity, acquired resistance, and loss of ERK-dependent negative feedback loops, which ultimately cause reactivation of ERK signaling through RTKs and WT KRAS (74, 75). Although there was some indication of clinical efficacy, clinical evaluation of the ERK-selective inhibitor ulixertinib in PDAC was terminated due to toxicity (103). Similarly, PI3K/AKT/mTOR pathway inhibitors alone or in combination with chemotherapy or other targeted inhibitors showed limited clinical success (82). Despite promising preclinical data using PDAC cell lines and GEMMs, these inhibitors did not demonstrate significant antitumor effects and/or were associated with dose-limiting toxicities in patients with PDAC (104).

Additionally, oncogenic KRAS effector signaling reprograms tumor metabolism in ways that could be exploited for therapeutic benefit (105). *KRAS*-mutant cancer cells exhibit increased glycolytic flux and increased dependency on glutamine metabolism and on nutrient-scavenging pathways such as autophagy and macropinocytosis, among others (106). These findings have sparked intense interest in targeting metabolic adaptations of *KRAS*-mutant PDAC, although so far with limited clinical benefit. Several clinical trials have been completed or are ongoing to target autophagy using hydroxychloroquine in combination with chemotherapy (NCT01978184) (107) or with MEK/ERK inhibitors in PDAC (NCT04386057, NCT03825289, NCT04132505) (108, 109). Devimistat (CPI-613), an inhibitor of the tricarboxylic acid cycle, has been evaluated in combination with modified FOLFIRINOX; however, it did not improve outcomes for patients with PDAC compared with chemotherapy alone (NCT01835041) (110). Devimistat is currently being evaluated as a triple combination with hydroxychloroquine and chemotherapy in patients with PDAC (NCT05733000). The glutaminase 1 inhibitor telaglenastat (CB-839) has shown limited clinical efficacy in advanced solid tumors when combined with PARP inhibitors (NCT03875313), perhaps owing to rapid metabolic adaptations that overcome glutamine dependency (111). Greater efficacy will require the development of more tolerable KRAS effector pathway inhibitors (or the use of direct KRAS inhibitors as discussed below) and more effective and selective inhibitors of metabolic pathways (112, 113).

## Development of direct KRAS inhibitors

### KRAS^G12C^ inhibitors.

KRAS has long been viewed as an “undruggable” target due to its high affinity for GTP (114) and the lack of suitable binding pockets for drug candidates (115). However, Shokat and colleagues challenged this notion in 2013 with the seminal discovery of a previously unseen switch II pocket that became visible only after being stabilized due to its occupancy by a small molecule covalently bound to the cysteine residue at the G12 position (116, 117). Just a few years after the initial discovery of the switch II pocket, two KRAS^G12C^ inhibitors, sotorasib (AMG 510) and adagrasib (MRTX849), entered clinical evaluation for *KRAS^G12C^*-mutant solid tumors (118, 119). The first clinical trial showed that sotorasib had an acceptable safety profile and demonstrated clinical benefit in patients with NSCLC, with an objective (or overall) response rate (ORR) of 37.1% and a median OS of 12.5 months (18, 120). The second clinical trial, of adagrasib, had similar outcomes in NSCLC, with an ORR of 42.9% and a median OS of 12.6 months (17).

Sotorasib and adagrasib were granted accelerated FDA approval for advanced NSCLC in 2021 and 2022, respectively. Furthermore, randomized, open-label phase III trial results demonstrated that the ORR was higher in patients with NSCLC treated with sotorasib compared with the standard-of-care docetaxel (28.1% and 13.2%, respectively), although the median OS was not significantly different between treatments (10.6 months for sotorasib and 11.3 months for docetaxel) (121). Similarly, the phase III trial comparing adagrasib with docetaxel found that the ORR was substantially higher in adagrasib-treated patients with NSCLC (31.9%) compared with docetaxel-treated patients (9.2%). Considering their selectivity for mutant over WT KRAS, these inhibitors caused unexpectedly high levels of treatment-related adverse events (TRAEs) of grade 3 or higher (33% for sotorasib [121], 47% for adagrasib [122]).

Although these initial findings sparked excitement in the *KRAS*-mutant cancer field, less than 2% of patients with PDAC harbor *KRAS^G12C^* mutations (Figure 1B), limiting how beneficial this therapeutic avenue might be. *KRAS^G12C^*-mutant patients with PDAC showed an ORR of 21.1% and median OS of 6.9 months without significant adverse events (123). Slightly better results were observed with adagrasib with ORR of 33.3% and median OS of 8 months (124) (Table 3). Although the response to KRAS^G12C^ inhibitors did not outperform the current standard of care, TRAEs were lower after KRAS^G12C^ inhibitor treatment compared with chemotherapy (7, 123).

**Figure 3:**
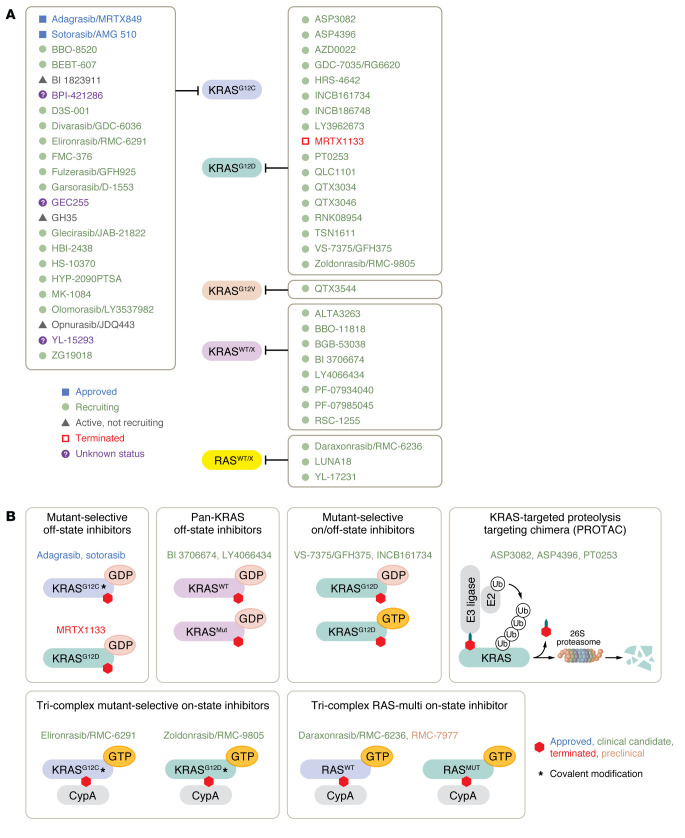
Direct KRAS inhibitors. (**A**) The current landscape of direct KRAS inhibitors and their status in preclinical and clinical stages. Blue indicates approved drugs, green indicates clinical trials that are recruiting, gray indicates active clinical trials that are not recruiting, red indicates terminated clinical trials, and purple indicates trials with unknown status. (**B**) The mechanisms of action of KRAS inhibitors are diverse. Mutant-selective inhibitors can be off-state, on-state, or off- and on-state inhibitors. Some inhibitors covalently modify mutant KRAS, others do not. There are multi-mutant or pan-KRAS and pan-RAS inhibitors that target WT and mutant KRAS/RAS proteins. Tri-complex inhibitors utilize cytosolic cyclophilin A (CypA) scaffold and KRAS degraders utilize ubiquitin-mediated proteasomal degradation of KRAS protein.

There are now over 20 additional direct KRAS^G12C^ inhibitors under clinical evaluation (Supplemental Table 1); the majority target GDP-bound KRAS^G12C^ and share a similar mechanism of action to sotorasib and adagrasib (Figure 3). Among these, divarasib has shown potentially superior activity versus the approved inhibitors and is currently in phase III evaluation compared directly with the two approved inhibitors (125). In contrast to the KRAS^G12C^(OFF) inhibitors, BBO-8520 is a first-in-class covalent KRAS^G12C^ inhibitor that binds to both GDP- and GTP-bound KRAS^G12C^ and is under phase I clinical evaluation in NSCLC (NCT06343402) (126). Additionally, RMC-4998 and its clinical analog elironrasib/RMC-6291 are members of a unique class of KRAS^G12C^ inhibitors, where the compound first forms a binary complex with a cytoplasmic chaperon cyclophilin A (CypA) and then binds to GTP-bound KRAS^G12C^, forming a tri-complex (127). Downstream KRAS signaling is inhibited because this tri-complex inhibitor sterically prevents effector interaction with KRAS. Elironrasib is also in phase I clinical trials for advanced KRAS^G12C^ solid tumors as a monotherapy (NCT05462717) and in combination with a multi-RAS inhibitor daraxonrasib/RMC-6236 (NCT06128551). There is also evidence to suggest that drug-modified KRAS^G12C^ oncoprotein fragments could harness an immune response. Recent proof-of-principle experiments suggested that ARS-1620- (128) or sotorasib-modified KRAS^G12C^ (129) are presented as neoantigens by class I MHC, which then recruit cytotoxic T cells to KRAS^G12C^ inhibitor-resistant cancer cells. It remains to be determined if any of the newer KRAS^G12C^ inhibitors will elicit stronger responses in *KRAS^G12C^*-mutant PDAC.

### KRAS^G12D^ inhibitors.

The substantial progress and success of KRAS^G12C^ inhibitors has stimulated intense efforts to develop inhibitors against other *KRAS* mutant proteins. This is of particular relevance to PDAC, where 41% of tumors are driven by *KRAS^G12D^* mutations (Figure 1B). The first KRAS^G12D^-selective inhibitor, MRTX1133, demonstrated near 1,000-fold selectivity for inhibiting KRAS^G12D^ signaling and *KRAS^G12D^*-mutant cancer cell growth as compared with KRAS WT (130, 131). MRTX1133 exhibited excellent antitumor efficacy and tumor regression, elicited an immune response in preclinical models, and entered phase I/II clinical evaluation for KRAS^G12D^ solid tumors in 2023 (130, 132). However, clinical evaluation of MRTX1133 (NCT0537706) was recently terminated because the drug exhibited high pharmacokinetic variability and failed to meet thresholds for advancement.

In contrast to MRTX1133, zoldonrasib/RMC-9805 is a covalent ON KRAS^G12D^-selective inhibitor. Zoldonrasib, a tri-complex inhibitor with CypA, binds to KRAS^G12D^ in its GTP-bound state and has demonstrated promising antitumor efficacy as both monotherapy and in combination with anti-PD1 therapy in preclinical KRAS^G12D^ models, including PDAC (133). Early clinical evaluation showed promising efficacy in PDAC (30% ORR) with very limited toxicity (Table 3). Several additional OFF (LY3962673, ref. 134, and QTX3046, ref. 135) and ON (GFH375/VS-7375, ref. 136; HRS-4642, ref. 137; TSN1611, ref. 138; and INCB161734, ref. 139) KRAS^G12D^-selective inhibitors are in phase I clinical evaluation (Supplemental Table 1).

### Pan-KRAS and multi-KRAS inhibitors.

Unlike the allele-selective KRAS inhibitors, pan-KRAS and multi-KRAS inhibitors inhibit multiple KRAS mutants as well as WT KRAS protein (Figure 3). Preclinical compounds BI-2865 and BI-2493 bind to a broad range of GDP-bound mutant KRAS proteins and WT KRAS but not WT HRAS or NRAS (140). In mice bearing *KRAS^G12C/D/V^* and *KRAS^A146V^* tumors, BI-2493 has demonstrated antitumor activity and inhibition of ERK phosphorylation without toxicity, as measured by changes in body weight. Although BI-2865 and BI-2493 are considered “pan-KRAS” inhibitors that target 18 of 24 most common KRAS mutations, they lack activity against KRAS^G12R^, KRAS^Q61L/K/R^, and KRAS^A59T^ mutant proteins (140). These compounds have also shown activity in *KRAS* WT-amplified tumors, most commonly seen in gastric and esophageal cancers (141). A clinical candidate BI 3706674 is now under clinical evaluation in cancers harboring *KRAS^G12V^* or *KRAS* WT amplifications (NCT06056024). QTX3034, a noncovalent multi-KRAS inhibitor against GDP-bound KRAS^G12D^ and to a lesser extent KRAS^G12V^ (142), is also under clinical evaluation as a monotherapy or in combination with cetuximab (NCT06227377) for patients with *KRAS^G12D^* solid tumors (Supplemental Table 1).

### Pan-RAS inhibitors.

Based on GEMM studies that observed deleterious consequences caused by genetic ablation of *Ras* genes (143–147), it was anticipated that a pan-RAS inhibitor would be toxic. Therefore, an unexpected and most encouraging clinical development in the field of direct KRAS inhibitors for PDAC treatment is the tri-complex, pan-RAS, ON selective inhibitors. RMC-7977 and its clinical analog daraxonrasib/RMC-6236 are first-in-class reversible tri-complex RAS(ON) pan-RAS-selective inhibitors that bind to both WT and mutant KRAS, NRAS, and HRAS proteins (148–150) (Figure 3). These inhibitors block RAS signaling by preventing effector binding and/or by stimulating intrinsic RAS GTPase activity (151). RMC-7977 treatment demonstrated potent inhibitory activity against a broad spectrum of *RAS* mutations, with *KRAS^G12X^*-mutant cancer cell lines displaying the highest degree of sensitivity. Furthermore, RMC-7977 caused robust and durable tumor suppression and multiple regressions in a large panel of *KRAS^G12X^* PDAC, CRC, and NSCLC xenograft models (148). Recent reports indicated that WT RAS and upstream RTK signaling limit the therapeutic efficacy of KRAS^G12C^ inhibitors (152). Due to its ability to bind and inhibit WT RAS proteins, RMC-7977 retained activity in KRAS^G12C^ inhibitor-resistant cancer cells (148).

Daraxonrasib is under clinical evaluation in *KRAS*-mutant solid tumors, including PDAC, with encouraging patient outcomes (NCT05379985). Preliminary reports from 42 patients with PDAC harboring *KRAS^G12X^* mutations demonstrated a median progression-free survival of 8.5 months and ORR of 27% (Table 3). Importantly, daraxonrasib was well tolerated, and the most common TRAEs were grade 1 or 2 rash, nausea, and vomiting (153). Recruitment for the RASolute 302 phase III clinical trial comparing daraxonrasib as a second-line treatment versus chemotherapy is currently ongoing (NCT06625320).

## Other KRAS therapeutic strategies

Although the field has been dominated by direct KRAS small-molecule inhibitors, several alternative anti-KRAS strategies, including RNAi, proteolysis targeting chimera (PROTAC), and immunotherapy-based approaches have been under clinical evaluation for *KRAS*-mutant solid tumors, including PDAC, albeit with less exciting results (Supplemental Table 1). siG12D-LODER is a novel bio-degradable polymeric matrix containing RNAi against KRAS^G12D/V^ that is implanted directly into the pancreas. Reports from a phase I/IIa clinical trial demonstrated that combination treatment with siG12D-LODER and chemotherapy was safe and well tolerated with a median OS of 15.1 months, although the current status of this RNAi therapy is unknown (NCT01676259) (154).

The development of PROTAC-based KRAS degraders is another emerging strategy for targeting *KRAS*-mutant cancers. ASP3082 is a PROTAC degrader that tags mutant KRAS^G12D^ for ubiquitin-mediated proteasomal degradation, with a strong selectivity for mutant KRAS protein over >9,000 other proteins. ASP3082 treatment decreased KRAS^G12D^ downstream signaling and cancer cell growth in vitro and in xenograft models after once-weekly intravenous administration (155). It is currently in phase I trials and has so far demonstrated an acceptable safety profile in patients with PDAC (NCT05382559) (156). Similarly, ACBI3 is a pan-KRAS PROTAC degrader active against 13 of 17 of the most common KRAS mutations; it demonstrated potent and durable inhibition of KRAS signaling in vitro and tumor regression in vivo (157). There are both advantages and disadvantages of utilizing PROTAC-based degraders compared with small-molecule inhibitors. Degraders might allow for targeting multiple KRAS mutations simultaneously and result in inhibition of all KRAS functions, not solely inhibition of downstream effector binding. However, due to their large molecular size, delivery of PROTACs is challenging and will require intravenous administration, compared with oral delivery of small molecules. It is also unknown if PROTAC degraders will be susceptible to the same or novel resistance mechanisms as small-molecule inhibitors.

Finally, several attempts have been made to use vaccines and T cell therapies to target KRAS. The TG01 vaccine, consisting of synthetic peptides against seven of the most common KRAS mutations, was used in combination with recombinant human GM-CSF. When given with gemcitabine, TG01 evoked an immune response and led to a median OS of 33.3 months, but it is no longer in active development (NCT202261714) (158). Furthermore, mRNA-5671/V94 (159), a lipid nanoparticle-based mRNA vaccine against several KRAS mutations (G12D, G12V, G13D, and G12C), was under clinical evaluation, but the trial has been terminated (NCT03948763). ELI-002 2P is a lymph node–targeted KRAS^G12D/G12R^ amphiphile vaccine. Early results demonstrated that ELI-002 2P elicited a notable T cell response without dose-limiting toxicities in patients with PDAC and CRC (NCT04853017) (160). KISIMA-02, another experimental approach under clinical investigation, is a three-component platform consisting of a vaccine against KRAS^G12D/G12V^ (ATP150/ATP152), a viral vector (VSV-GP154), and the immune checkpoint inhibitor ezabenlimab (161) (NCT05846516). Recently, trial results (NCT03592888) from a mature dendritic cell vaccine against KRAS^MUT^ (mDC3/8-KRAS) were published, which reported a KRAS^G12V^-specific T cell response in vaccinated individuals (162). There is also preliminary evidence that individualized mRNA neoantigen vaccines administered in combination with anti–PD-L1 inhibitors and chemotherapy in surgery-eligible patients with PDAC elicited T cell responses and correlated with delayed recurrence (NCT04161755) (163). Harnessing the immune system as an anti-KRAS therapy has the potential for long-lasting benefits; however, it has so far been largely unsuccessful in the clinic and requires better understanding of the immunosuppressive tumor microenvironment of PDAC.

## Resistance to KRAS inhibitors and promising combination strategies

**Figure 4:**
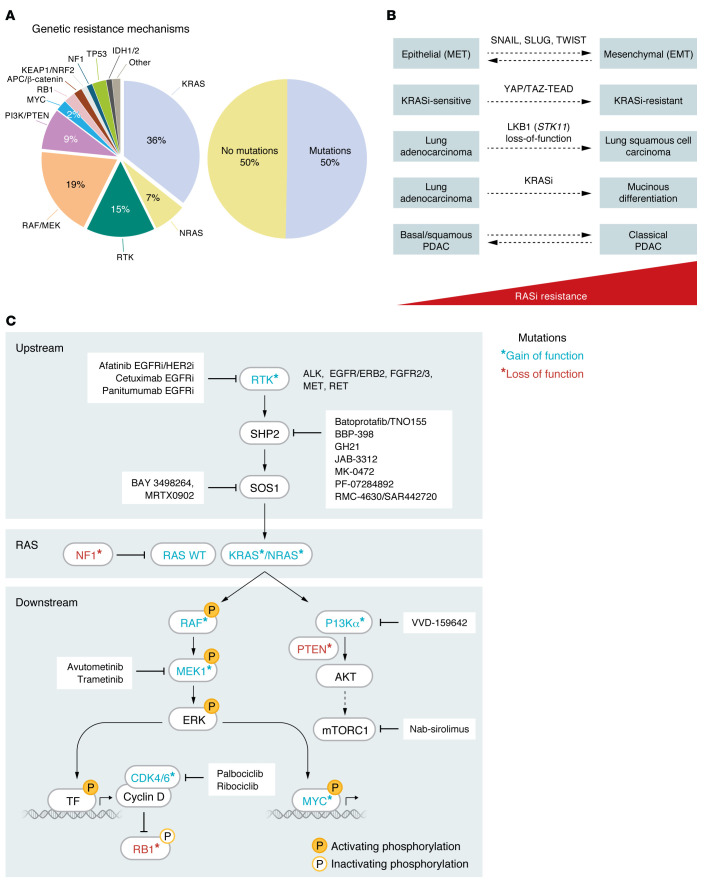
Resistance mechanisms to KRAS^G12C^ inhibitors and combination strategies. (**A**) Sequencing of circulating tumor DNA from patients who relapsed on adagrasib, sotorasib, divarasib, or LY3537982 treatment demonstrated that genetic alterations occurred at the level of RAS or in the upstream and downstream components of RAS signaling. RAS-level alterations included mutations and/or amplifications in *KRAS* and *NRAS* and mutations in *NF1*. Upstream signaling alterations included mutations, amplifications, and fusions in RTKs. Downstream signaling alterations included mutational activation of downstream ERK MAPK and PI3K effector signaling components, amplification of *MYC*, etc. No genetic mutations were found in 50% of patients who relapsed on KRAS^G12C^ treatment. (**B**) Most combination strategies with KRAS inhibitors are based on resistance mechanisms that have been identified in relapsed patients and in preclinical studies that include signal transduction and kinase inhibitors, among others (Tables 3 and 4). (**C**) Nongenetic mechanisms driving resistance to KRAS inhibitors may include transcriptional reprogramming, changes in cellular states (epithelial to mesenchymal [EMT], adeno-to-squamous carcinoma, or adenocarcinoma to mucinous differentiation), and/or changes in molecular subtypes. MET,mesenchymal to epithelial transition; RASi, RAS inhibitor.

The challenge of nearly all targeted therapies is primary (innate) and acquired resistance. Our understanding of resistance mechanisms to KRAS inhibitors in PDAC remains limited and stems primarily from patients with NSCLC, CRC, and PDAC who have been treated with KRAS^G12C^-selective inhibitors (87, 88, 164–167). Unlike the resistance to protein kinase inhibitors, which commonly arises due to second site mutations that impair inhibitor binding, putative KRAS inhibitor resistance mechanisms are varied and complex (Figure 4A). Strikingly, up to a dozen distinct mutations have been found within one patient. Targeted DNA sequencing analyses of circulating tumor DNA in patients experiencing relapse have identified genetic alterations at three distinct levels that ultimately converge to reactivate KRAS signaling. These alterations occurred at the level of RAS itself, in the components upstream of RAS, or in the effectors downstream of RAS (87, 88, 164–169) (Figure 4B). In addition, mutations in components outside the RAS signaling network have also been described (Supplemental Figure 1). Nongenetic mechanisms of resistance were found in half of patients who relapsed on KRAS^G12C^ inhibitor treatment (Figure 4C). Some of these include transcriptional reprogramming that changes cellular states such as epithelial-to-mesenchymal transition (164), activation of YAP/TAZ signaling (167), adeno-to-squamous cell carcinoma transition (87), and mucinous differentiation (170).

The emerging complex resistance mechanisms suggest that combination strategies will be essential to improve the depth and duration of response to KRAS inhibitors. Guided in part by genetic alterations associated with relapsed tumors, and by preclinical CRISPR genetic screens or experimentally induced resistance assays, multiple combinations with KRAS inhibitors are currently under clinical evaluation (Figure 4B, Tables 3 and 4, and Supplemental Table 1). To date, the most promising combinations have involved inhibitors of upstream RTKs, particularly EGFR. The combination of anti-EGFR monoclonal antibodies with adagrasib and sotorasib led to the approval of these combinations for *KRAS^G12C^*-mutant CRC (171, 172). Other combinations with inhibitors of additional components of the RAS signaling network (e.g., SOS1, ref. 173), immune checkpoint inhibitors (e.g., anti–PD-1 monoclonal antibodies, ref. 174), standard-of-care chemotherapy (e.g., gemcitabine/nab-paclitaxel, ref. 164), and co-occurring genetic alterations (*MTAP*-deletions with PRMT5 inhibitors, ref. 175) are currently under evaluation (Tables 3 and 4 and Supplemental Table 1).

## Conclusions and future directions

After nearly four decades of effort, where many initially promising ideas failed to deliver clinically effective anti-KRAS therapies, the shattering of the myth that KRAS is undruggable has brought exciting new optimism that KRAS inhibitors will finally provide a significant therapeutic breakthrough in the treatment of PDAC. It is now conceivable that KRAS inhibitors may replace ineffective cytotoxic drugs as the standard of care. However, rather than marking the end of the road for anti-KRAS drug discovery, it is clearly early days in the process. The “best” class of KRAS inhibitors remains to be determined: mutation-selective, pan-KRAS or pan-RAS, allosteric small molecules versus degraders. The complex nature of mechanisms of resistance is arguably the most daunting challenge, highlighting the importance of continuing to develop other therapeutic strategies beyond direct KRAS inhibitors as well as to identify multiple effective combination therapies. Additional mutation-selective strategies, in particular for G12R and Q61X patients, may be needed. Biomarkers to identify patients who will respond to KRAS inhibitors and to monitor the efficacy of target inhibition will also be important.

The history of anti-KRAS drug discovery has been marked by misconceptions and an incomplete understanding of KRAS function, where concepts once considered dogma were later smashed and replaced. Although KRAS is one of the most intensely studied oncogenes, much remains to be understood about how it functions as a cancer driver. Despite these challenges, the discovery of KRAS inhibitors has ushered in a time of cautious optimism that the upward rate of pancreatic cancer deaths and the incremental steps in improvements to the 5-year survival rate of PDAC may soon be in our past.

## Supplementary Material

Supplemental data

## Figures and Tables

**Table 4:**
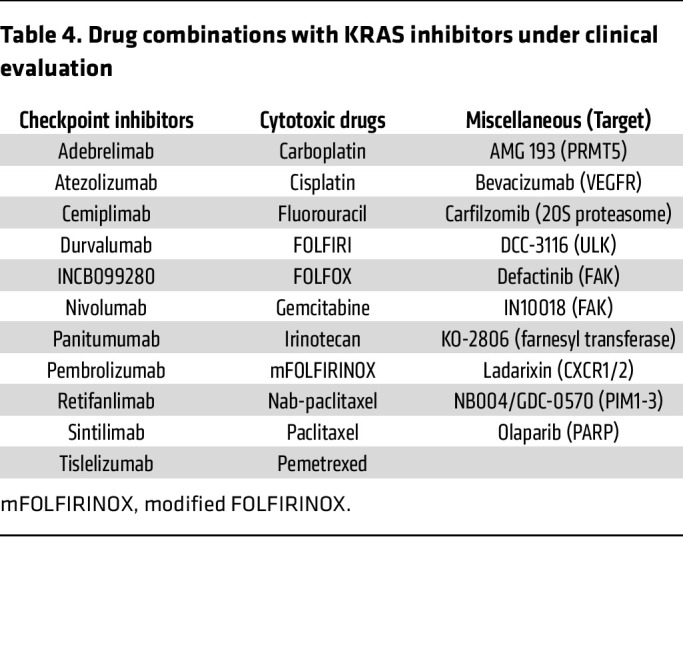
Drug combinations with KRAS inhibitors under clinical evaluation

**Table 1:**
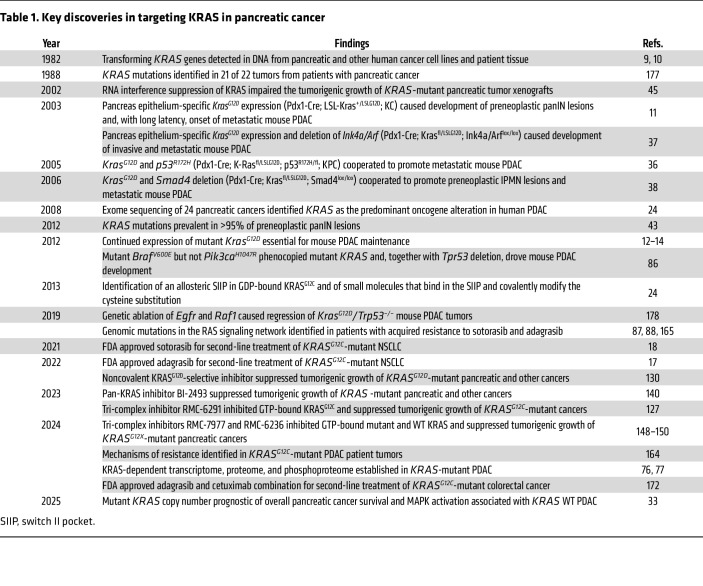
Key discoveries in targeting KRAS in pancreatic cancer

**Table 2:**
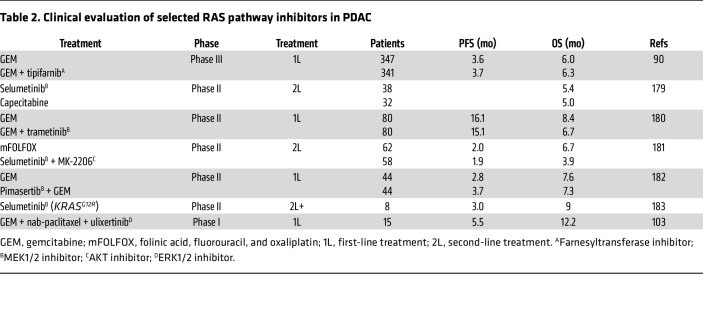
Clinical evaluation of selected RAS pathway inhibitors in PDAC

**Table 3:**
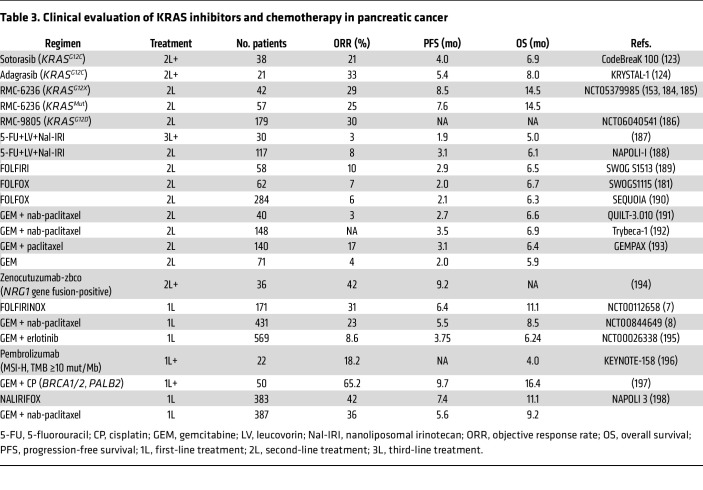
Clinical evaluation of KRAS inhibitors and chemotherapy in pancreatic cancer

## Version 1 08/15/2025

Electronic publication
