# Supplementary material for: KRAS: the Achilles’ heel of pancreas cancer biology
Source: J Clin Invest. 2025 Aug 15;135(16):e191939. doi: 10.1172/JCI191939 (PMC12352898; doi:10.1172/JCI191939)
Supplement: Supplemental data [file jci-135-191939-s255.pdf]

Supplemental Table 1. Clinical evaluation of direct RAS inhibitors

Name	Target	Sponsor	ClinicalTrials.gov ID	Official title[a]	References
**G12C-selective**					
Adagrasib/MRTX849/BMS-986503	G12C	Mirati Therapeutics/ Bristol Myers Squibb	NCT03785249	Phase 1/2 Study of MRTX849 in Patients With Cancer Having a KRAS G12C Mutation KRYSTAL-1 (in combination with PD-1 inhibitor pembrolizumab, EGFR inhibitor cetuximab, EGFR/HER2 inhibitor afatinib)	(1-3)
			NCT04330664	Adagrasib in Combination With TNO155 (SHP2 inhibitor) in Patients With Cancer (KRYSTAL 2)	
			NCT04613596	Phase 2 Trial of Adagrasib Monotherapy and in Combination With Pembrolizumab (PD-1 inhibitor) and a Phase 3 Trial of Adagrasib in Combination in Patients With a KRAS G12C Mutation (KRYSTAL-7) [first-line treatment]	
			NCT04685135	Phase 3 Study of MRTX849 (Adagrasib) vs Docetaxel in Patients With Advanced Non-Small Cell Lung Cancer With KRAS G12C Mutation	
			NCT04793958	Phase 3 Study of MRTX849 With Cetuximab (EGFR inhibitor) vs Chemotherapy in Patients With Advanced Colorectal Cancer With KRAS G12C Mutation (KRYSTAL-10)	
			NCT04975256	Adagrasib in Combination With BI 1701963 (S0S1 inhibitor) in Patients With Cancer (KRYSTAL 14)	
			NCT05178888	Adagrasib in Combination With Palbociclib (CDK4/6 inhibitor) in Patients With Advanced Solid Tumors (KRYSTAL-16)	
			NCT05263986	The Clinical Trial to Evaluate the Pharmacokinetics and Safety of MRTX849 in Patients With Advanced Solid Tumors	
			NCT05375994	Study of Avutometinib [RAF-MEK inhibitor VS-6766] + Adagrasib in KRAS G12C NSCLC Patients	
			NCT05472623	Neoadjuvant KRAS G12C Directed Therapy With Adagrasib (MRTX849) With or Without Nivolumab (PD-1 inhibitor)	
			NCT05578092	A Phase 1/2 Study of MRTX0902 (SOS1 inhibitor alone or with adagrasib) in Solid Tumors With Mutations in the KRAS MAPK Pathway	
			NCT05609578	Combination Therapies [pembrolizumab + pemetrexed + cisplatin/carboplatin] With Adagrasib in Patients With Advanced NSCLC With KRAS G12C Mutation	
			NCT05634525	Phase Ib Trial of the KRASG12C Inhibitor Adagrasib (MRTX849) in KRAS G12C Mutant Metastatic Pancreatic Cancer Patients	
			NCT05673187	Adagrasib in Patients With KRASG12C-mutant NSCLC Who Are Elderly or Have Poor Performance Status	

			NCT05722327	Phase I Trial of Adagrasib (MRTX849) in Combination With Cetuximab (EGFR inhibitor) and Irinotecan (topoisomerase I inhibitor) in Patients With Colorectal Cancer	
			NCT05840510	Adagrasib in Combination With Nab-Sirolimus (mTOR inhibitor) in Patients With Advanced Solid Tumors and Non-Small Cell Lung Cancer With a KRAS G12C Mutation (KRYSTAL -19)	
			NCT05853575	Trial of Two Adagrasib Dosing Regimens in NSCLC With KRAS G12C Mutation (KRYSTAL 21)	
			NCT06026410	KO-2806 (Farnesyltransferase inhibitor) Monotherapy and Combination Therapies (with adagrasib or cabozantinib) in Advanced Solid Tumors	
			NCT06039384	A Study of INCB099280 (PD-L1 inhibitor) in Combination With Adagrasib in Adults With Advanced Solid Tumors Harboring a KRASG12C Mutation	
			NCT06130254	Phase Ib Trial of the KRAS G12C Inhibitor Adagrasib (MRTX849) in Combination With the PARP Inhibitor Olaparib in Patients With KRAS G12C Mutated Advanced Solid Tumors, With a Focus on Gynecological, Breast, Pancreatic and KEAP1 Mutated Non-small Cell Lung Cancers	
			NCT06248606	Adagrasib + SRS (stereotactic radiosurgery) for Patients With Metastatic KRAS G12C-mutated NSCLC With Untreated Brain Metastases	
			NCT06412198	A Multicenter Phase 1b/2 Study of Adagrasib, Cetuximab, and Cemiplimab for Metastatic Colorectal Cancer Harboring KRAS G12C Mutations	
			NCT06764771	A Phase 1/1b Open-label Study of BMS-986488 as Monotherapy and Combination [adagrasib, cetuximab] Therapy in Participants With Advanced Malignant Tumors	
			NCT06875310	A Randomized, Double-Blind, Phase 3 Trial of Adagrasib Plus Pembrolizumab Plus Chemotherapy [carboplatin, cisplatin, pemetrexed] vs. Placebo Plus Pembrolizumab Plus Chemotherapy in Participants With Previously Untreated, Locally Advanced or Metastatic Non-squamous Non-small Cell Lung Cancer With KRAS G12C Mutation (KRYSTAL-4)	
Sotorasib/AMG 510	G12C	Amgen	NCT03600883	A Phase 1/2, Study Evaluating the Safety, Tolerability, PK, and Efficacy of Sotorasib (AMG 510) in Subjects With Solid Tumors With a Specific KRAS Mutation (CodeBreaK 100)	(4-6)
			NCT04092673	Study of eFT226 in Subjects With Selected Advanced Solid Tumor Malignancies	
			NCT04185883	Sotorasib Activity in Subjects With Advanced Solid Tumors With KRAS p.G12C Mutation (CodeBreak 101)	

			NCT04303780	Study to Compare AMG 510 "Proposed INN Sotorasib" With Docetaxel in Non Small Cell Lung Cancer (NSCLC) (CodeBreak 200)	
			NCT04380753	AMG 510 Ethnic Sensitivity Study (CodeBreaK 105)	
			NCT04625647	Testing the Use of Targeted Treatment (AMG 510) for KRAS G12C Mutated Advanced Non-squamous Non-small Cell Lung Cancer (A Lung-MAP Treatment Trial)	
			NCT04720976	JAB-3312 (SHP2 inhibitor) Based Combination Therapy in Adult Patients With Advanced Solid Tumors	
			NCT04887064	Pharmacokinetics of Sotorasib in Healthy Participants and Participants With Moderate or Severe Hepatic Impairment	
			NCT04892017	A Phase 1/2 Study of DCC-3116 [ULK1/2 inhibitor] (in combination with MEKi trametinib, MEKi binimetinib or G12Ci sotorasib) in Patients With RAS/MAPK Pathway Mutant Solid Tumors	
			NCT04933695	A Study of Sotorasib (AMG 510) in Participants With Stage IV NSCLC Whose Tumors Harbor a KRAS p.G12C Mutation in Need of First-line Treatment	
			NCT04959981	A Study of Anti-Cancer Therapies Targeting the MAPK Pathway in Patients With Advanced NSCLC	
			NCT05036291	A Study of NB004 [PIM kinase inhibitor] as Monotherapy or Combination Therapy (with sotorasib) in Patients With Advanced Solid Tumors	
			NCT05054725	Combination Study of RMC-4630 [SH2 inhibitor] and Sotorasib for NSCLC Subjects With KRASG12C Mutation After Failure of Prior Standard Therapies	
			NCT05074810	Phase 1/2 Study of Avutometinib [VS-6766; RAF-MEK inhibitor] + Sotorasib With or Without Defactinib [FAK inhibitor] in KRAS G12C NSCLC Patients	
			NCT05118854	A Phase II Study of Neoadjuvant Sotorasib in Combination With Cisplatin or Carboplatin and Pemetrexed for Surgically Resectable Stage IIA-IIIB Non-Squamous Non-Small Cell Lung Cancer With a KRAS p.G12C Mutation	
			NCT05198934	Sotorasib and (EGFRi) Panitumumab Versus Investigator's Choice for Participants With Kirsten Rat Sarcoma (KRAS) p.G12C Mutation	
			NCT05272423	Studying Pathways of Resistance in KRAS-driven Cancers	
			NCT05273047	RW Efficacy of Sotorasib in KRAS G12C-mutated Metastatic NSCLC	
			NCT05311709	Sotorasib in Advanced KRASG12C-mutated Non-small Cell Lung Cancer Patients With Comorbidities	

			NCT05398094	Clinical Trial of AMG510 in Stage III Unresectable NSCLC KRAS p.G12C Patients and Ineligible for Chemo-radiotherapy	
			NCT05451056	An Open-label, Phase 2trial of Sotorasib in KRAS G12C-mutant Non-small-cell Lung Cancer (NSCLC) Patients and a Translational Study to Find Acquired Resistance Mechanism to Sotorasib	
			NCT05480865	SHP2 Inhibitor BBP-398 in Combination With Sotorasib in Patients With Advanced Solid Tumors and a KRAS-G12C Mutation	
			NCT05564377	Targeted Therapy Directed by Genetic Testing in Treating Patients With Locally Advanced or Advanced Solid Tumors, The ComboMATCH Screening Trial	
			NCT05631249	Sotorasib in Previously Treated Locally Advanced or Metastatic NSCLC Subjects With Mutated KRAS p.G12C	
			NCT05638295	Testing the Use of AMG 510 [Sotorasib] and Panitumumab [EGFR inhibitor] as a Targeted Treatment for KRAS G12C Mutant Solid Tumor Cancers (A ComboMATCH Treatment Trial)	
			NCT05815173	Ladarixin [CXCR1/2 inhibitor] With Sotorasib in Advanced NSCLC	
			NCT05845450	Pre-operative Targeted Treatments in Molecularly Selected Resectable Colorectal Cancer (UNICORN) (pMMR/MSS status with sotorasib + EGFR inhibitor panitumumab)	
			NCT05920356	A Study Evaluating Sotorasib Platinum Doublet Combination [carboplatin and pemetrexed] Versus Pembrolizumab Platinum Doublet Combination as a Front-Line Therapy in Participants With Stage IV or Advanced Stage IIIB/C Nonsquamous Non-Small Cell Lung Cancers (CodeBreaK 202)	
			NCT05993455	A Phase 2 Basket Trial in Which Patients With Advanced Solid Tumors Carrying the KRAS G12C Mutation Receive Treatment With a Combination of Sotorasib and Panitumumab [EGFR inhibitor]	
			NCT06127940	K-SAB Trial - Sotorasib Followed by SBRT (stereotactic radiation therapy) to 1-3 Lesions in Advanced NSCLC With KRASG12C Mutation	
			NCT06249282	Carfilzomib (proteasome inhibitor) in Combination With Sotorasib for the Treatment of Patients With KRAS G12C Mutated Advanced or Metastatic Non-small Cell Lung Cancer	
			NCT06252649	Phase 3 Multicenter, Randomized, Open-label, Active-controlled Study of Sotorasib, Panitumumab and FOLFIRI Versus FOLFIRI With or Without Bevacizumab-awwb for	

				Treatment-naïve Subjects With Metastatic Colorectal Cancer With KRAS p.G12C Mutation (CodeBreaK 301)	
			NCT06314763	Rivaroxaban Sotorasib Interaction Study	
			NCT06333678	A Study Comparing Sotorasib With Durvalumab (PD-L1 inhibitor) in People With Non-Small Cell Lung Cancer (NSCLC)	
			NCT06333951	AMG 193 (PRMT5 inhibitor) Alone or in Combination With Other Therapies in Subjects With Advanced Thoracic Tumors With Homozygous MTAP-deletion (Master Protocol)	
			NCT06582771	A Study of Sotorasib in People With Non-Small Cell Lung Cancer	
			NCT06659341	Phase 1 Study of a SOS1 Inhibitor, BAY 3498264, in Combination in Participants With Advanced KRASG12C-mutated Solid Tumors	
			NCT06804824	A Phase 1/1b, Open-Label, Multicenter, First-in-Human Dose Escalation and Dose Expansion Study to Evaluate the Safety, Tolerability, Pharmacokinetics, Pharmacodynamics, and Anti-Tumor Activity of VVD-159642, a RAS-PI3Kα Inhibitor, as a Single Agent and in Combination [+ sotorasib or trametinib] in Participants With Advanced Solid Tumors	
			NCT06807619	BrainMet ADePPT (Anticancer Drug Penetration Platform Trial)	
			NCT06892054	Sotorasib Combined With First-line Chemotherapy [gem/nab-P or FOLFIRINOX] for Advanced Pancreatic Adenocarcinoma With KRAS p.G12C Mutation	
BBO-8520	G12C	BridgeBio	NCT06343402	A Phase 1a/1b Open-Label Study of BBO-8520 Monotherapy and BBO-8520 in Combination With Pembrolizumab in Subjects With Advanced KRASG12C Mutant Non-Small Cell Lung Cancer - the ONKORAS-101 Study	(7)
BEBT-607	G12C	BeBetter Med	NCT06117371	A Two-phase, Multicenter, Open Phase I Study of BEBT-607 Tablets in The Treatment of Advanced or Metastatic Solid Tumors With KRAS G12C Mutation	
BI 1823911	G12C	Boehringer Ingelheim	NCT04973163	A Phase Ia/Ib, Open-label, Multicentre Dose-escalation and Expansion Study to Investigate the Safety, Pharmacokinetics and Preliminary Efficacy of BI 1823911 as a Monotherapy and in Combination With Other Anti-cancer Therapies in Patients With Advanced or Metastatic Solid Tumours Expressing KRAS G12C Mutation [active, not recruiting]	(8, 9)
BPI-421286	G12C	Betta Pharmaceuticals	NCT05315180	A Phase 1, Open-label Study Evaluating the Safety, Tolerability, Pharmacokinetics, and Efficacy of BPI-421286 in Subjects With Advanced Solid Tumors [unknown status]	(10)

D3S-001	G12C	D3 Bio (Wuxi)	NCT05410145	A Phase 1/2, Open Label, Dose-escalation, and Dose-expansion Study Evaluating the Safety, Tolerability, Pharmacokinetics, Pharmacodynamics, and Preliminary Efficacy of D3S 001 Monotherapy or Combination [PD-1 inhibitor pembrolizumab or carboplatin plus pemetrexed] Therapy in Subjects with Advanced Solid Tumors with a KRAS P.G12C Mutation	(11)
Divarasib/GDC-6036	G12C	Roche/Genentech	NCT03178552	A Phase II/III Multicenter Study Evaluating the Efficacy and Safety of Multiple Targeted Therapies as Treatments for Patients With Advanced or Metastatic Non-Small Cell Lung Cancer (NSCLC) Harboring Actionable Somatic Mutations Detected in Blood (B-FAST: Blood-First Assay Screening Trial) [active, not recruiting]	(12-14)
			NCT04302025	NAUTIKA1: Multicenter, Phase II, Neoadjuvant and Adjuvant Study of Multiple Therapies in Biomarker-Selected Patients With Resectable Stages IB-III Non-Small Cell Lung Cancer	
			NCT04449874	A Phase Ia/Ib Dose-Escalation and Dose-Expansion Study Evaluating the Safety, Pharmacokinetics, and Activity of GDC-6036 as a Single Agent and in Combination With Other Anti-cancer Therapies [atezolizumab, cetuximab] in Patients With Advanced or Metastatic Solid Tumors With a KRAS G12C Mutation	
			NCT04589845	Tumor-Agnostic Precision Immunooncology and Somatic Targeting Rational for You (TAPISTRY) Phase II Platform Trial	
			NCT04929223	A Phase I/Ib Global, Multicenter, Open-label Umbrella Study Evaluating the Safety and Efficacy of Targeted Therapies in Subpopulations of Patients With Metastatic Colorectal Cancer (INTRINSIC)	
			NCT05789082	A Phase Ib/II, Open-Label, Multicenter Study Evaluating the Safety, Activity, and Pharmacokinetics of Divarasib in Combination With Other Anti-Cancer Therapies (PD-1 inhibitor pembrolizumab alone or pembrolizumab + carboplatin or cisplatin) in Patients With Previously Untreated Advanced Or Metastatic Non-Small Cell Lung Cancer With a KRAS G12C Mutation	
			NCT06497556	A Phase III, Randomized, Open-Label, Multicenter Study Evaluating the Efficacy and Safety of Divarasib Versus Sotorasib or Adagrasib in Patients With Previously Treated KRAS G12C-Positive Advanced or Metastatic Non-Small Cell Lung Cancer	
			NCT06793215	A Phase III, Randomized, Open-Label Study Evaluating the Efficacy and Safety of Divarasib and Pembrolizumab Versus	

				Pembrolizumab and Pemetrexed and Carboplatin or Cisplatin in Patients With Previously Untreated, KRAS G12C-Mutated, Advanced or Metastatic Non-Squamous Non-Small Cell Lung Cancer [not yet recruiting]	
Elironrasib/RMC-6291	G12C	Revolution Medicines	NCT05462717	Phase 1/1b, Multicenter, Open-Label, Dose Escalation and Dose Expansion Study of RMC-6291 Monotherapy in Subjects With Advanced KRASG12C Mutant Solid Tumors [active, not recruiting]	(15)
			NCT06128551	Phase 1b, Multicenter, Open-Label, Dose Escalation and Dose Expansion Study of RMC-6291 in Combination With RMC-6236 (RAS-multi inhibitor) in Participants With Advanced KRAS G12C Mutant Solid Tumors	
			NCT06162221	A Platform Study of RAS(ON) Inhibitor Combinations [RMC-6291 +/- RMC-6236 + pembrolizumab +/- cisplatin, carboplatin, pemetrexed] in Patients with RAS-Mutated Non-Small Cell Lung Cancer (NSCLC)	
FMC-376	G12C	Frontier Medicines	NCT06244771	An Open-Label, Phase 1/2 Dose Escalation, Dose Expansion and Cohort Expansion Study Evaluating the Safety, PK and Clinical Activity of FMC-376 in Participants With KRAS G12C Mutated Locally Advanced Unresectable or Metastatic Solid Tumors	(16, 17)
Fulzerasib/IBI351/GFH925	G12C	Innovent Biologics	NCT05005234	An Open-label, Multi-center Phase I/II Clinical Study Evaluating the Safety/Tolerability, Pharmacokinetics, and Effectiveness of GFH925 in Patients With Advanced Solid Tumors With KRAS G12C Mutations	(18, 19)
			NCT05497336	An Open-label, Multicenter, Phase Ib/III Study of Efficacy and Safety of IBI351 in Combination With (EGFRi) Cetuximab in Subjects With KRAS G12C Mutated Metastatic Colorectal Cancer	
			NCT05504278	An Open-label, Multi-center Phase Ib/III Study Evaluating the Efficacy and Safety of IBI351 in Combination With Sintilimab [PD-1 inhibitor] ± Chemotherapy [IBI351 + Sintilimab + pemetrexed, or IBI351 + Sintilimab + pemetrexed + cisplatin/carboplatin] in Advanced Non-squamous Non-small Cell Lung Cancer Subjects With KRAS G12C Mutation	
			NCT05756153	A Phase Ib/II, Multi-Center, Open-Label Study to Evaluate the Safety/Tolerability, Pharmacokinetics, and Efficacy of GFH925 in Combination with Cetuximab in Previously Untreated Advanced NSCLC Harboring KRAS G12C Mutation	
Garsorasib/D-1553	G12C	InventisBio	NCT04585035	A Phase 1/2, Open Label Study to Evaluate the Safety, Tolerability, Pharmacokinetics and Efficacy of D-1553 in	(20-22)

				Subjects With Advanced or Metastatic Solid Tumors With KRasG12C Mutation [active, not recruiting]	
			NCT05379946	A Phase 1/2, Open Label Study to Evaluate the Safety, Tolerability, Pharmacokinetics and Efficacy of D-1553 in Combination With IN10018 (FAK inhibitor) in Subjects With Advanced or Metastatic Solid Tumors With KRasG12C Mutation	
			NCT05383898	A Phase 1/2, Open-Label Study to Evaluate the Safety, Tolerability, Pharmacokinetics and Efficacy of D-1553 in Subjects With Advanced or Metastatic Solid Tumors [completed]	
			NCT05492045	A Phase 1b/2 Study to Evaluate the Safety, Tolerability, Pharmacokinetics and Efficacy of D-1553 Combination (with standard treatment for NSCLC) Therapy in Subjects With KRAS G12C-Mutated Locally Advanced or Metastatic Non-Small Cell Lung Cancer [terminated]	
			NCT06166836	A Phase 1b/II, Open-label Study to Evaluate the Safety, Tolerability, Pharmacokinetics and Efficacy of D-1553 Combined With IN10018 [FAK inhibitor] in Subjects With Locally Advanced or Metastatic Solid Tumors With KRAS G12C Mutation	
			NCT06300177	A Randomized, Controlled, Double-blind, Double-simulated, Multicenter Phase III Clinical Study Evaluating D-1553 Tablet Versus Docetaxel Injection for KRAS G12C Mutation-positive Locally Advanced or Metastatic Non-small Cell Lung Cancer After Prior Standard Therapy Failure	
			NCT06435455	A Phase Ib/II Clinical Study Evaluating the Safety, Tolerability, Pharmacokinetics and Efficacy of GH21 [SHP2 inhibitor] Capsule Combined With D-1553 Tablets in Patients With Locally Advanced or Metastatic Solid Tumors Harboring KRAS G12C Mutation [not yet recruiting]	
GEC255	G12C	GenEros Biopharma	NCT05768321	A Phase 1 Open-Label Study to Assess the Safety, Pharmacokinetics, and Preliminary Efficacy of GEC255 Oral Tablets in Subjects With Advanced Solid Tumors With KRAS p.G12C Mutation [Unknown status]	(23)
GH35	G12C	Suzhou Genhouse Bio	NCT05010694	A Phase I Study Evaluating the Safety, Tolerability, Pharmacokinetic Characteristics, and Primary Antitumor Activity of GH35 in Patients With Advanced Solid Tumors With KRAS Mutation [active, not recruiting]	
Glecirasib/JAB-21822	G12C	Jacobio	NCT05002270	A Phase 1/2, Multi-Center, Open-Label Study to Evaluate the Safety, Tolerability, Pharmacokinetics, and Preliminary Evidence of Antitumor Activity of JAB-21822 Monotherapy and	(24-28)

				Combination Therapy [+ cetuximab] in Adult Patients With Advanced Solid Tumors Harboring KRAS G12C Mutation	
			NCT05009329	Multi-center, Open, Dose-escalation, and Expanded Phase I/II Clinical Study to Evaluate the Safety, Tolerability, Pharmacokinetics, and Antitumor Activity of JAB-21822 in Advanced Solid Tumors With KRAS p.G12C Mutation [active, not recruiting]	
			NCT05194995	A Phase Ib/II Trial of JAB-21822 in Combination With Cetuximab in Patients With Advanced Colorectal Cancer, Small Intestine Cancer and Appendiceal Cancer With KRAS G12C Mutation [active, not recruiting]	
			NCT05276726	A Phase Ib/II, Single Arm, Multi-Center, Open Label Study to Evaluate the Safety, Tolerability, Pharmacokinetics, and Preliminary Evidence of JAB-21822 in Advanced or Metastatic Non-small Cell Lung Cancer With a KRAS p.G12C and STK11 Co-mutation and Wild-type KEAP1	
			NCT05288205	A Phase 1/2a Clinical Study to Evaluate the Safety, Tolerability, Pharmacokinetics and Antitumor Activity of JAB-21822 in Combination With JAB-3312 [SHP2 inhibitor] in Patients With Advanced Solid Tumors Harboring KRAS p.G12C Mutation	
			NCT06008288	A Phase 2, Multi-Center, Open-Label, Single-arm Study to Evaluate the Efficacy and Safety of JAB-21822 Monotherapy in Patients With Locally Advanced or Metastatic KRAS p.G12C Mutated Pancreatic Cancer	
			NCT06416410	An Open-label, Randomized, Positive Control, Multicenter Phase III Clinical Study. Evaluating JAB-21822 Combined With JAB-3312 [SHP2 inhibitor] Compared Tislelizumab Combined With Pemetrexed + Carboplatin in the First Line for Treatment of Advanced Non-squamous Non-small Cell Lung Cancer With KRAS p.G12C Mutation	
			NCT06563999	Neoadjuvant Umbrella Trial Directed by Next Generation Sequencing for Patients With Unresectable Stage III NSCLC Harboring Rare Mutations (Without EGFR Sensitizing Mutations)	
			NCT06838338	A Phase Ib, Open Label, Clinical Trial of JAB-21822 Combined With Second Line Chemotherapy for Metastatic Colorectal Cancer With KRAS G12C Mutation	
HBI-2438	G12C	HUYABIO	NCT05485974	A Phase 1, Open Label, Dose Escalation of HBI-2438 in Patients With Advanced Malignant Solid Tumors Harboring KRAS G12C Mutation	

HS-10370	G12C	Jiangsu Hansoh	NCT05367778	A Phase 1/2, Open-label, Multicenter Study Evaluating the Safety, Tolerability, Pharmacokinetics, and Efficacy of HS-10370 Monotherapy in Patients With Advanced Solid Tumors	(29)
			NCT06594874	A Phase Ib Study Evaluating the Safety, Tolerability, Pharmacokinetics and Activity of HS-10370 in Addition to Other Anti-cancer Therapies [PD-L1 inhibitor adebrelimab, cisplatin, carboplatin, pemetrexed] in Participants with KRAS G12C Mutation Advanced Solid Tumors [not yet recruiting]	
			NCT06963502	A Phase Ib Study Evaluating the Safety, Tolerability, Pharmacokinetics, Activity and Immunogenicity of HS-10370 in Addition to Other Anti-cancer Therapies [adebrelimab, capecitabine, oxaliplatin, FU, irinotecan, cisplatin, carboplatin] in Patients With Advanced Solid Tumors	
HYP-2090PTSA	G12C	Sichuan Huiyu	NCT06243354	An Open-label, Multi-center, Multi-cohort, Phase 1/2 Study to Evaluate the Safety, Tolerability, Pharmacokinetics and Efficacy of HYP-2090PTSA in Patients With Advanced Solid Tumors Harboring KRAS Mutation	
MK-1084	G12C	Merck	NCT05067283	A Phase 1, Open-Label, Multicenter Study to Assess Safety, Tolerability, PK, and Efficacy of MK-1084 as Monotherapy and in Combination With Pembrolizumab in Subjects With KRAS G12C Mutant Advanced Solid Tumors	(30-32)
			NCT05853367	A Phase 1/1b Open-label, Multicenter Clinical Study of MK-0472 [SHP2 inhibitor) as Monotherapy and Combination (pembrolizumab/MK-1084) Therapy in Participants With Advanced/Metastatic Solid Tumors	
			NCT06345729	A Phase 3, Randomized, Double-blind, Multicenter Study of MK-1084 in Combination With Pembrolizumab Compared With Pembrolizumab Plus Placebo as Firstline Treatment of Participants With KRAS G12C-Mutant, Metastatic NSCLC With PD-L1 TPS ≥50%	
			NCT06997497	A Phase 3, Randomized, Open-label, Multicenter Clinical Study to Evaluate the Safety and Efficacy of MK-1084, Cetuximab, and mFOLFOX6 Versus mFOLFOX6 With or Without Bevacizumab as First-line Treatment of Participants With KRAS G12C-mutant, Locally Advanced Unresectable or Metastatic Colorectal Cancer (KANDLELIT-012)	
Olomorasib/LY3537982	G12C	Eli Lilly	NCT04956640	A Phase 1/2 Study of LY3537982 in Patients With KRAS G12C-Mutant Advanced Solid Tumors [monotherapy and in combination with PD-1 inhibitor pembrolizumab, EGFRi cetuximab, pemetrexed, cisplatin, carboplatin)	(33-37)
			NCT06119581	SUNRAY-01, A Global Pivotal Study in Participants With KRAS G12C-Mutant, Locally Advanced or Metastatic Non-	

				Small Cell Lung Cancer Comparing First-Line Treatment of LY3537982 and (PD-1 inhibitor) Pembrolizumab vs Placebo and Pembrolizumab in Those With PD-L1 Expression ≥50% or LY3537982 and Pembrolizumab, Pemetrexed, Platinum vs Placebo and Pembrolizumab, Pemetrexed, Platinum Regardless of PD-L1 Expression	
			NCT06235983	An Open-label, Single-arm, Multicenter, Phase 1 Study to Investigate the Pharmacokinetics, Safety, Tolerability, and Antitumor Activity of LY3537982 in Chinese Patients With KRAS G12C-Mutant Advanced Solid Tumors [active, not recruiting]	
			NCT06890598	A Phase 3, Multicenter, Double-Blind, Placebo-controlled Study Assessing the Efficacy and Safety of Olomorasib in Combination With Standard of Care Immunotherapy [pembrolizumab, durvalumab] in Participants With Resected or Unresectable KRAS G12C-Mutant, Non-Small Cell Lung Cancer - SUNRAY-02	
Opnurasib/JDQ443	G12C	Novartis	NCT04699188	A Phase Ib/II [KontRASt-01] Open-label, Multi-center Dose Escalation Study of JDQ443 in Patients With Advanced Solid Tumors Harboring the KRAS G12C Mutation [active, not recruiting]	(38-40)
			NCT05132075	A Randomized, Controlled, Open Label, Phase III Study Evaluating the Efficacy and Safety of JDQ443 Versus Docetaxel in Previously Treated Subjects With Locally Advanced or Metastatic KRAS G12C Mutant Non-small Cell Lung Cancer [active, not recruiting]	
			NCT05358249	KontRASt-03: A Phase Ib/II, Multicenter, Open-label Platform Study of JDQ443 With Select Combinations [trametinib, ribociclib, cetuximab] in Patients With Advanced Solid Tumors Harboring the KRAS G12C Mutation [active, not recruiting]	
			NCT05445843	KontRASt-06: An Open-label Phase II Trial Evaluating the Activity and Safety of JDQ443 Single-agent as First-line Treatment for Patients With Locally Advanced or Metastatic KRAS G12C-mutated Non-small Cell Lung Cancer With a PD-L1 Expression < 1% or a PD-L1 Expression ≥ 1% and an STK11 Co-mutation [active, not recruiting]	
			NCT05714891	Neoadjuvant Platform Trial in Patients With Surgically Resectable Non-Small Cell Lung Cancer (NSCLC) [active, not recruiting]	
			NCT05999357	A Phase II Study evaluating intracranial efficacy of JDQ443 in patients With KRAS G12C+ NSCLC and brain Metastases [withdrawn]	

YL-15293	G12C	Shanghai YingLi	NCT05173805	Phase I Clinical Study on the Safety, Tolerance, Pharmacokinetics and Efficacy of YL-15293 in Patients With Advanced Solid Tumor With KRAS Mutation [unknown status]	
			NCT05119933	A Phase 1/2, Study Evaluating the Safety, Tolerability, Pharmacokinetics, and Preliminary Anti-Tumor Activity of YL-15293 in Subjects With Advanced Solid Tumors With a KRAS G12C Mutation [unknown status]	
ZG19018	G12C	Suzhou Zelgen	NCT06237400	A Phase I/II Dose Escalation and Expansion Study to Evaluating the Tolerability, Safety, Efficacy, and Pharmacokinetics of ZG19018 in Patients With KRAS G12C Mutant Advanced Solid Tumors.	(41, 42)
**G12D-selective**					
ASP3082	G12D	Astellas	NCT05382559	A Phase 1 Study of ASP3082 in Participants With Previously Treated Locally Advanced or Metastatic Solid Tumor Malignancies With KRAS G12D Mutation	(43-46)
ASP4396	G12D	Astellas Pharma	NCT06364696	An Open-label Phase 1 Study of ASP4396 in Participants With Locally Advanced (Unresectable) or Metastatic Solid Tumor Malignancies With KRAS G12D Mutation	(47)
AZD0022	G12D	AstraZeneca	NCT06599502	A Phase I/IIa, Open-label, Multi-centre Study to Assess the Safety, Tolerability, Pharmacokinetics, and Preliminary Efficacy of AZD0022 Monotherapy and in Combination With Anti-cancer Agents [cetuximab] in Participants With Tumours Harbouring a KRASG12D Mutation (ALAFOSS-01)	(48)
GDC-7035/RG6620	G12D	Roche/Genentech	NCT06619587	A Phase I/II Dose-Escalation and Expansion Study Evaluating the Safety, Pharmacokinetics, and Activity of GDC-7035 as a Single Agent and in Combination [not indicated] With Other Anti-Cancer Therapies in Patients With Advanced Solid Tumors With a KRAS G12D Mutation	
GFH375/VS-7375	G12D	Genfleet	NCT06500676	An Open-Label, Phase I/II Clinical Study to Evaluate the Safety, Tolerability, Pharmacokinetics, and Efficacy of GFH375 in Patients With KRAS G12D Mutant Advanced Solid Tumors	(49, 50)
		Verastem	NCT07020221	A Phase 1/2a, Open-label Study of VS-7375, a KRAS G12D (ON/OFF) Inhibitor, as Monotherapy and in Combination, in Patients With Advanced KRAS G12D-Mutated Solid Tumors	
		Genfleet	NCT07026916	A Single-Arm, Multicenter, Open-Label, Phase II Clinical Study to Evaluate the Efficacy, Safety/Tolerability, and Pharmacokinetic Characteristics of GFH375 Monotherapy in Patients With Previously Treated KRAS G12D Mutant Metastatic Pancreatic Cancer	
HRS-4642	G12D	Jiangsu HengRui	NCT05533463	Phase I Study to Evaluate the Safety ,Tolerability, and Pharmacokinetics of HRS-4642 in Patients With Advanced Solid Tumors Harboring KRAS G12D Mutation	(51, 52)

			NCT06385678	A Phase IB/II Clinical Study on the Safety, Tolerability and Efficacy of HRS-4642 in Combination (PD-L1 inhibitor adebrelimab, pemetrexed, or SHR-A1921 Trop-2-targeted topoisomerase I inhibitor ADC) With Anti-tumor Medication in Subjects With Advanced Solid Tumors [enrolled by invitation]	
			NCT06427239	A Single-center, Open-label, Exploratory Study of HRS-4642 Combined With Adebrelimab [PD-L1 inhibitor] in the Treatment of Advanced Pancreatic Cancer	
			NCT06520488	Phase IB/II Clinical Study of the Safety, Tolerability and Efficacy of HRS-4642 in Combination With Anti-tumor Agents [SHR-A1904*] in Subjects With Advanced Solid Tumors [not yet recruiting]<br>*ADC: CLDN18.2 mAb and topoisomerase I inhibitor payload [not yet recruiting]	
			NCT06547736	A Single-center, Open-label, Exploratory Platform Research on Precision Therapy of Advanced Pancreatic Cancer [HRS-4642 + SHR-A2102*/SHR-A1904/SHR-A1811**]<br>* SHR-A2102 ADC nectin-4 mAb and topoisomerase I inhibitor payload<br>** SHR-A1811 ADC HER2 mAb and DNA topoisomerase I inhibitor	
			NCT06587061	An Exploratory Clinical Study of HRS-4642 in Combination With Gemcitabine and Albumin-bound Paclitaxel for the Neoadjuvant and Adjuvant Treatment of Pancreatic Cancer [not yet recruiting]	
			NCT06620848	A Study of HRS-4642 Monotherapy or in Combination with Adebrelimab [PD-L1 inhibitor] in Patients with Advanced Biliary Tract Tumors. [not yet recruiting]	
			NCT06770452	A Single-arm Phase II Study of HRS-4642 with Nimotuzumab [anti-EGFR] and Chemotherapy [gemcitabine + nab-paclitaxel] for First-line Treatment of Advanced Pancreatic Cancer Patients with KRAS G12D Mutations [not yet recruiting]	
			NCT06773130	An Exploratory Clinical Study of HRS-4642 Combined With Nimotuzumab in the Treatment of Recurrent or Metastatic Pancreatic Ductal Adenocarcinoma [not yet recruiting]	
			NCT06938282	An Exploratory Clinical Study of HRS-4642 in Combination With Immunotherapy and Chemotherapy for Pancreatic Cancer	
			NCT06955390	An Exploratory Clinical Study of HRS-4642 in Combination With SHR-A2102 [ADC comprised of mAb against nectin-4, and a topoisomerase I inhibitor] for the Treatment of Advanced Solid Tumors	

INCB161734	G12D	Incyte	NCT06179160	A Phase 1, Open-Label, Multicenter Study of INCB161734 [monotherapy and in combination with EGFR inhibitor cetuximab and PD-1 inhibitor retifanlimab] in Participants With Advanced or Metastatic Solid Tumors With KRAS G12D Mutation	(53)
INCB186748	G12D	Incyte	NCT06818812	A Phase 1, Open-Label, Multicenter Study of INCB186748 in Participants With Advanced or Metastatic Solid Tumors With KRAS G12D Mutation	
LY3962673	G12D	Eli Lilly	NCT06586515	A Phase 1a/1b Trial of LY3962673 (monotherapy and in combination with EGFR inhibitor cetuximab or chemotherapy: gemcitabine, nab-paclitaxel, oxaliplatin, leucovorin, irinotecan, 5-flurouracil) in Participants With KRAS G12D-Mutant Solid Tumors	(54-56)
MRTX1133/BMS-986508	G12D	Mirati Therapeutics / Bristol Myers Squibb	NCT05737706	A Phase 1/2 Multiple Expansion Cohort Trial of MRTX1133 in Patients With Advanced Solid Tumors Harboring a KRAS G12D Mutation [terminated]	(57-59)
PT0253	G12D	PAQ Therapeutics	NCT06797336	A Phase 1, Open-Label Dose Escalation and Expansion Study of PT0253 in Participants with KRAS G12D Mutated Advanced Solid Tumors	
QLC1101	G12D	Qilu Pharmaceutical Co., Ltd.	NCT06403735	A Phase I Clinical Study to Evaluate the Safety, Tolerability, Pharmacokinetics, and Preliminary Efficacy of QLC1101 Monotherapy in the Treatment of Patients With Advanced Solid Tumors Harboring a KRAS G12D Mutation	
			NCT06949761	A Phase Ib/II Clinical Study to Evaluate the Safety, Tolerability, and Efficacy of QLC1101 in Combination With Other Therapies [QL1203 EGFR mAb, QL2107 PD-1 mAb, QL1706 PD-1 and CTLA-4 mAb, docetaxel] in the Treatment of Patients With Advanced Solid Tumors Harboring a KRAS G12D Mutation	
QTX3034	G12D	Quanta Therapeutics	NCT06227377	A Phase 1 Trial Evaluating the Safety, Tolerability, PK, and Efficacy of QTX3034 in Patients With Solid Tumors With KRASG12D [in combination with cetuximab] Mutation	(60-62)
QTX3046	G12D-preferring multi-KRAS inhibitor	Quanta Therapeutics	NCT06428500	A Phase 1 Trial Evaluating the Safety, Tolerability, Pharmacokinetics and Efficacy of QTX3046]monotherapy or in combination with EGFRi cetuximab] in Patients With Advanced Solid Tumors With KRAS G12D Mutations	(63)
RNK08954	G12D	Ranok Therapeutics	NCT06667544	A Phase 1/2, First-in-Human, Open-label Study Evaluating the Safety, Tolerability, Pharmacokinetics, and Efficacy of RNK08954 in Patients With Advanced Solid Tumors With a KRAS G12D Mutation TRIAD1 (Trial of RNK08954 In KRAS G12D Mutation)	

TSN1611	G12D	Tyligand Bioscience	NCT06385925	Phase 1/2 Study of TSN1611 in Subjects With Advanced Solid Tumors Harboring KRAS G12D Mutation	(64)
Zoldonrasib/RMC-9805	G12D	Revolution Medicines	NCT06040541	Phase 1/1b, Multicenter, Open-Label, Study of RMC 9805 in Participants With Advanced KRASG12D-Mutant Solid Tumors (+/- RMC-6236)	(65-67)
			NCT06162221	A Platform Study of RAS(ON) Inhibitor Combinations [RMC-9805 G12Di +/- RMC-6236 + pembrolizumab +/- cisplatin, carboplatin, pemetrexed] in Patients with RAS-Mutated Non-Small Cell Lung Cancer (NSCLC)	
			NCT06445062	A Platform Study of RAS(ON) Inhibitors [RMC-6236 +/- RMC-9805] in Patients With Gastrointestinal Solid Tumors [+ FU-based regimens (PDAC/CRC)/EGFR inhibitor cetuximab +/- mFOLFIRINOX (PDAC/CRC)/gemcitabine + nab-paclitaxel (PDAC)]	
			NCT06922591	A Phase 1/2, Multicenter, Open-Label Study to Evaluate Safety, Tolerability & Antitumor Activity of TNG462 in Combination With Other Agents [RMC-9805] in Patients With Pancreatic or Non-Small Cell Lung Cancer With MTAP Loss & RAS Mutation	
**G12V-selective**					
QTX3544	G12V-preferring multi-KRAS inhibitor	Quanta Therapeutics	NCT06715124	A Phase 1 Trial Evaluating the Safety, Tolerability, Pharmacokinetics and Preliminary Anti-Tumor Activity of QTX3544 in Patients With Advanced Solid Tumors With KRAS G12V Mutations	(68)
**Pan-KRAS**					
ALTA3263	KRAS WT and mutant	Alterome Therapeutics	NCT06835569	A Phase 1/1b Multiple Cohort Trial of ALTA3263 in Patients with Advanced Solid Tumors with KRAS Mutations	(69)
BBO-11818	KRAS WT and mutant	BridgeBio	NCT06917079	A Phase 1a/1b Open-Label Study Evaluating the Safety, Tolerability, Pharmacokinetics, and Efficacy of BBO-11818 in Subjects With Advanced KRAS Mutant Cancers	(70)
BGB-53038	KRAS WT and mutant	BeiGene	NCT06585488	A Phase 1a/1b Study to Investigate the Safety, Tolerability, Pharmacokinetics, Pharmacodynamics, and Preliminary Antitumor Activity of BGB-53038, a Pan-KRAS Inhibitor, as Monotherapy or in Combinations [tislelizumab anti- PD-1, cetuximab anti-EGFR] in Patients With Advanced or Metastatic Solid Tumors With KRAS Mutations or Amplifications	
BI 3706674	KRAS WT and mutant	Boehringer Ingelheim	NCT06056024	Open-label Dose-finding Trial to Explore Safety, Pharmacokinetics, Pharmacodynamics, and Efficacy of BI 3706674 Given Orally as Monotherapy in Patients With Unresectable Metastatic KRAS Wild Type Amplified Gastric,	(71)

				Oesophageal, and Gastroesophageal Junction Adenocarcinoma	
LY4066434	KRAS WT and mutant	Eli Lilly	NCT06607185	A Phase 1a/1b Study of the Pan-KRAS Inhibitor LY4066434 in Participants With KRAS Mutant Solid Tumors (monotherapy or in combination with EGFRi cetuximab and chemotherapy: nab-paclitaxel, gemcitabine, oxaliplatin, leucovorin, irinotecan, 5-fluorouracil, carboplatin, cisplatin, pemetrexed, or PD-1 inhibitor pembrolizumab)	(72)
PF-07934040	KRAS WT and mutant	Pfizer	NCT06447662	A Phase 1 Open-Label Study of PF-07934040 as a Single Agent and in Combination With Other Targeted Agents [EGFRi cetuximab, VEGFi bevacizumab, PD-1 inhibitor pembrolizumab, gemcitabine, paclitaxel, nab-paclitaxel, cisplatin, carboplatin, oxaliplatin, fluorouracil, leucovorin, pemetrexed] in Participants With Advanced Solid Tumors Harboring Mutations in the KRAS Gene	
PF-07985045	RAS WT and mutant	Pfizer	NCT06704724	A Phase 1 Open-Label Study of PF-07985045 as A Single-Agent and in Combination With Other Anti-Cancer Agents [gemcitabine + nab-paclitaxel, cetuximab, FOLFOX, bevacizumab, pembrolizumab, pemetrexed, cisplatin, paclitaxel, carboplatin, PF-07284892 (SHP2 inhibitor)], in Participants With Advanced Solid Tumors	
RSC-1255	RAS WT and mutant	RasCal Therapeutics	NCT04678648	A Phase Ia/Ib, Open Label, Multi-center, Non-randomized Dose Escalation and Dose Expansion Study of RSC-1255 in Patients With Advanced Solid Tumor Malignancies	
**Pan-RAS**					
Daraxonrasib/RMC-6236	RAS WT and mutant	Revolution Medicines	NCT05379985	A Multicenter Open-Label Study of RMC-6236 in Patients With Advanced Solid Tumors Harboring Specific Mutations in RAS	(73-75)
			NCT06040541	Phase 1/1b, Multicenter, Open-Label, Study of RMC 9805 [G12D inhibitor] in Participants With Advanced KRASG12D-Mutant Solid Tumors	
			NCT06128551	Phase 1b, Multicenter, Open-Label, Dose Escalation and Dose Expansion Study of RMC-6291 [G12C inhibitor] in Combination With RMC-6236 in Participants With Advanced KRAS G12C Mutant Solid Tumors	
			NCT06162221	A Platform Study of RAS(ON) Inhibitor Combinations [RMC-6291 G12Ci or RMC-9805 G12Di +/- RMC-6236 + pembrolizumab +/- cisplatin, carboplatin, pemetrexed] in Patients with RAS-Mutated Non-Small Cell Lung Cancer (NSCLC)	
			NCT06445062	A Platform Study of RAS(ON) Inhibitors [RMC-6236 +/- RMC-9805] in Patients With Gastrointestinal Solid Tumors [+ FU-based regimens (PDAC/CRC)/EGFR inhibitor cetuximab +/-	

				mFOLFIRINOX (PDAC/CRC)/gemcitabine + nab-paclitaxel (PDAC)]	
			NCT06625320	RASolute 302: a Phase 3 Multicenter, Open-label, Randomized Study of RMC-6236 Versus Investigator's Choice of Standard of Care Therapy in Patients with Previously Treated [2L] Metastatic Pancreatic Ductal Adenocarcinoma (PDAC)	
			NCT06881784	RASolve 301: Phase 3 Multicenter, Open Label, Randomized Study of RMC-6236 Versus Docetaxel in Patients With Previously Treated Locally Advanced or Metastatic RAS[MUT] NSCLC	
			NCT06922591	A Phase 1/2, Multicenter, Open-Label Study to Evaluate Safety, Tolerability & Antitumor Activity of TNG462 in Combination With Other Agents [RMC-6236] in Patients With Pancreatic or Non-Small Cell Lung Cancer With MTAP Loss & RAS Mutation	
LUNA18	RAS WT and mutant	Chugai Pharmaceutical	NCT05012618	A Phase 1 Open-label, Dose-escalation and Cohort Expansion Study of LUNA18 Monotherapy and Combination Therapy in Patients With Locally Advanced or Metastatic Solid Tumors	(76)
YL-17231/TEB-17231	RAS WT and mutant	280 Bio	NCT06078800	Phase I Clinical Study on the Safety, Tolerance, Pharmacokinetics and Efficacy of Pan-KRAS Inhibitor YL-17231 in Patients With Advanced Solid Tumors With KRAS Mutation	(77)
			NCT06096974	A Multi-Center, Open-Label, Phase I Study to Evaluate the Safety, Tolerability, Pharmacokinetics and Preliminary Anti-tumor Activity of Pan-RAS Inhibitor YL-17231 in Patients With Advanced Solid Tumors Harboring Mutations in KRAS, HRAS, or NRAS	
**Other KRAS inhibitor strategies**					
ATP150/ATP152	G12D/G12V (Vaccine)	Amal Therapeutics	NCT05846516	A Phase 1b Study to Evaluate the Safety, Tolerability and Preliminary Efficacy of ATP150/ATP152, VSV-GP154 and Ezabenlimab (BI 754091) in Patients With KRAS G12D/G12V Mutated Pancreatic Ductal Adenocarcinoma (KISIMA-02)	
ABO2102-001	KRAS mutant (Vaccine)	Ruijin Hospital	NCT06577532	A Clinical Study to Investigate Safety, Tolerability, Immunogenicity, and Preliminary Anti-Tumor Activity of KRAS Neoantigen mRNA Vaccine (ABO2102) in Participants With KRAS-mutated Advanced Pancreatic Cancer	
ChanghaiH-PP06	KRAS G12V (T cell therapy)	Changhai Hospital	NCT04146298	Clinical Trial Evaluating the Safety and Activity of Mutant KRAS G12V-specific TCR Transduced T Cell Therapy for Advanced Pancreatic Cancer	
ELI-002	KRAS mutant (Vaccine)	Elicio Therapeutics	NCT04853017	First in Human Phase 1 Trial of ELI-002 Immunotherapy as Treatment for Subjects With Kirsten Rat Sarcoma (KRAS)	(78)

				Mutated Pancreatic Ductal Adenocarcinoma and Other Solid Tumors [active, not recruiting]	
J1994/KRAS Peptide Vaccine	KRAS mutant (Vaccine)	Sidney Kimmel Comprehensive Cancer Center at Johns Hopkins	NCT04117087	Pooled Mutant KRAS-Targeted Long Peptide Vaccine Combined With Nivolumab and Ipilimumab for Patients With Resected MMR-p Colorectal and Pancreatic Cancer	
J2456/KRAS Vaccine with Poly-ICLC adjuvant	KRAS mutant (Vaccine)	Sidney Kimmel Comprehensive Cancer Center at Johns Hopkins	NCT06411691	Pooled Mutant KRAS-Targeted Long Peptide Vaccine Combined With Balstilimab and Botensilimab for Patients With Stage IV MMR-p Colorectal Cancer and Pancreatic Ductal Adenocarcinoma	
NT-112	KRAS G12D (T cell therapy)	AstraZeneca	NCT06218914	An Open-label, Phase 1, Multicenter Study to Evaluate the Safety and Preliminary Anti-tumor Activity of NT-112 in Human Leukocyte Antigen-C*08:02-Positive Adult Subjects With Unresectable, Advanced, and/or Metastatic Solid Tumors That Are Positive for the KRAS G12D Mutation	
Targovax TG-01/Stimulon QS-21/KRAS Vaccine	KRAS mutant (Vaccine)	Georgetown University	NCT06015724	A Phase 2 Study Evaluating the Efficacy of Anti-CD38 Antibody in Combination With KRAS Vaccine and Anti-PD-1 Antibody in Subjects With Pancreatic Ductal Adenocarcinoma and Refractory Non-Small Cell Lung Cancer	
[a]Additional comments added in bracketed text					

References

1. Hallin J, et al. The KRAS^{G12C} inhibitor MRTX849 provides insight toward therapeutic susceptibility of KRAS-mutant cancers in mouse models and patients. *Cancer Discov.* 2020;10(1):54-71.
2. Janne PA, et al. Adagrasib in non-small-cell lung cancer harboring a KRAS^{G12C} mutation. *N Engl J Med.* 2022;387(2):120-131.
3. Yaeger R, et al. Efficacy and safety of adagrasib plus cetuximab in patients with KRAS^{G12C}-mutated metastatic colorectal cancer. *Cancer Discov.* 2024;14(6):982-993.
4. Canon J, et al. The clinical KRAS^{G12C} inhibitor AMG 510 drives anti-tumour immunity. *Nature.* 2019;575(7781):217-223.
5. Skoulidis F, et al. Sotorasib for lung cancers with KRAS p.G12C mutation. *N Engl J Med.* 2021;384(25):2371-2381.
6. Fakih MG, et al. Sotorasib plus panitumumab in refractory colorectal cancer with mutated KRAS^{G12C}. *N Engl J Med.* 2023;389(23):2125-2139.
7. Maciag AE, et al. Discovery of BBO-8520, a first-in-class direct and covalent dual inhibitor of GTP-bound (ON) and GDP-bound (OFF) KRASG12C. *Cancer Discov.* 2025;15(3):578-594.
8. Savarese F, et al. Abstract 1271: In vitro and in vivo characterization of BI 1823911 - a novel KRAS^{G12C} selective small molecule inhibitor. *Cancer Res.* 2021;81(13_Supplement):1271-1271.
9. Waizenegger IC, et al. Abstract 2667: Trial in progress: Phase 1 study of BI 1823911, an irreversible KRAS^{G12C} inhibitor targeting KRAS in its GDP-loaded state, as monotherapy and in combination with the pan-KRAS SOS1 inhibitor BI 1701963 in solid tumors expressing KRAS^{G12C} mutation. *Cancer Res.* 2022;82(12_Supplement):2667-2667.
10. Zhu X, et al. Abstract 5443: BPI-421286: A highly potent small molecule inhibitor targeting KRAS^{G12C} mutation. *Cancer Res.* 2022;82(12_Supplement):5443-5443.
11. Zhang J, et al. D3S-001, a KRAS^{G12C} inhibitor with rapid target engagement kinetics, overcomes nucleotide cycling and demonstrates robust preclinical and clinical activities. *Cancer Discov.* 2024.
12. Sacher A, et al. Single-agent divarasib (GDC-6036) in solid tumors with a KRAS^{G12C} mutation. *N Engl J Med.* 2023;389(8):710-721.
13. Purkey H. Abstract ND11: Discovery of GDC-6036, a clinical stage treatment for KRAS^{G12C}-positive cancers. *Cancer Res.* 2022;82(12_Supplement):ND11-ND11.

14. Weiss A, et al. Discovery, preclinical characterization, and early clinical activity of JDQ443, a structurally novel, potent, and selective covalent oral inhibitor of $KRAS^{G12C}$. *Cancer Discov.* 2022;12(6):1500-1517.
15. Jänne PA, et al. Abstract PR014: Preliminary safety and anti-tumor activity of RMC-6291, a first-in-class, tri-complex $KRAS^{G12C}$ (ON) inhibitor, in patients with or without prior $KRAS^{G12C}$ (OFF) inhibitor treatment. *Mol Cancer Ther.* 2023;22(12_Supplement):PR014-PR014.
16. Keating AT, et al. Phase 1/2 study of FMC-376 an oral $KRAS^{G12C}$ dual inhibitor in participants with locally advanced unresectable or metastatic solid tumors (PROSPER). *J Clin Oncol.* 2024;42(16_suppl):TPS3184-TPS3184.
17. Patel S, et al. Abstract 1142: Discovery of FMC-376 a novel orally bioavailable inhibitor of activated $KRAS^{G12C}$. *Cancer Res.* 2023;83(7_Supplement):1142-1142.
18. Gregorc V, et al. Krocus: A phase II study investigating the efficacy and safety of fulzerasib (GFH925) in combination with cetuximab in patients with previously untreated advanced kras g12c mutated nsclc. *J Clin Oncol.* 2024;42(17_suppl):LBA8511-LBA8511.
19. Majem M, et al. Lba1: First-line (1l) fulzerasib + cetuximab in $KRAS^{G12C}$ advanced NSCLC: Updated efficacy and safety from KROCUS study. *J Thorac Oncol.* 2025;20(3):S1.
20. Li Z, et al. D-1553 (garsorasib), a potent and selective inhibitor of $KRAS^{G12C}$ in patients with NSCLC: Phase 1 study results. *J Thorac Oncol.* 2023;18(7):940-951.
21. Xu RH, et al. 550o safety and efficacy of D-1553 in combination with cetuximab in KRAS G12C mutated colorectal cancer (CRC): A phase II study. *Ann Oncol.* 2023;34:S410-S411.
22. Li Z, et al. Abstract CT246: Open-label, single-arm, multicenter, phase 2 trial of garsorasib in KRAS G12C-mutated non-small-cell lung cancer. *Cancer Res.* 2024;84(7_Supplement):CT246-CT246.
23. Zhang Y, et al. Phase 1 study evaluating the safety, tolerability, pharmacokinetics (PK), and efficacy of GEC255, a novel $KRAS^{G12C}$ inhibitor, in advanced solid tumors. *J Clin Oncol.* 2023;41(16_suppl):9112-9112.
24. Li J, et al. A phase I/II study of first-in-human trial of JAB-21822 ($KRAS^{G12C}$ inhibitor) in advanced solid tumors. *J Clin Oncol.* 2022;40(16_suppl):3089-3089.
25. Wang P, et al. 30P investigation of $KRAS^{G12C}$ inhibitor JAB-21822 as a single agent and in combination with SHP2 inhibitor JAB-3312 in preclinical cancer models. *Ann Oncol.* 2022;33:S1441.
26. Li J, et al. Preliminary activity and safety results of $KRAS^{G12C}$ inhibitor glecirasib (JAB-21822) in patients with pancreatic cancer and other solid tumors. *J Clin Oncol.* 2024;42(3_suppl):604-604.
27. Zhao J, et al. Updated safety and efficacy data of combined $KRAS^{G12C}$ inhibitor (glecirasib, JAB-21822) and SHP2 inhibitor (JAB-3312) in patients with *KRAS p.G12C* mutated solid tumors. *J Clin Oncol.* 2024;42(16_suppl):3008-3008.
28. Shi Y, et al. A pivotal phase 2 single-arm study of glecirasib (JAB-21822) in patients with NSCLC harboring $KRAS^{G12C}$ mutation. *J Clin Oncol.* 2024;42(36_suppl):468214-468214.
29. Dong X, et al. Abstract ct119: Safety and efficacy of HS-10370 in $KRAS^{G12C}$-mutated solid tumors including non-small cell lung cancer (NSCLC). *Cancer Res.* 2024;84(7_Supplement):CT119-CT119.
30. Rojas C, et al. 663p safety and preliminary efficacy of the $KRAS^{G12C}$ inhibitor MK-1084 in solid tumors and in combination with pembrolizumab in NSCLC. *Ann Oncol.* 2023;34:S466-S467.
31. Cobb PW, et al. 1392TiP Phase III study of pembrolizumab plus MK-1084 vs pembrolizumab plus placebo as first-line treatment for metastatic non-small cell lung cancer (NSCLC) with a $KRAS^{G12C}$ mutation and PD-L1 tumour proportion score (TPS) ≥ 50%: MK-1084-004. *Ann Oncol.* 2024;35:S871-S872.
32. Sacher AG, et al. MK-1084 for $KRAS^{G12C}$-mutated (mut) metastatic non–small-cell lung cancer (mNSCLC): Results from KANDLELIT-001. *J Clin Oncol.* 2025;43(16_suppl):8605-8605.
33. Burns TF, et al. Efficacy and safety of olomorasib (LY3537982), a second-generation $KRAS^{G12C}$ inhibitor (G12Ci), in combination with pembrolizumab in patients with KRAS G12C-mutant advanced NSCLC. *J Clin Oncol.* 2024;42(16_suppl):8510-8510.
34. Heist RS, et al. Pan-tumor activity of olomorasib (LY3537982), a second-generation $KRAS^{G12C}$ inhibitor (G12Ci), in patients with $KRAS^{G12C}$-mutant advanced solid tumors. *J Clin Oncol.* 2024;42(16_suppl):3007-3007.
35. Negrao MV, et al. SUNRAY-01, a pivotal, global study of olomorasib (LY3537982) in combination with pembrolizumab with or without chemotherapy for 1L treatment in KRAS G12C-mutant advanced NSCLC. *J Clin Oncol.* 2024;42(16_suppl):TPS8649-TPS8649.
36. Hollebecque A, et al. Efficacy and safety of LY3537982, a potent and highly selective $KRAS^{G12C}$ inhibitor in $KRAS^{G12C}$-mutant GI cancers: Results from a phase 1 study. *J Clin Oncol.* 2024;42(3_suppl):94-94.

37. Fujiwara Y, et al. OA14.04 efficacy and safety of olomorasib with pembrolizumab + chemotherapy as first-line treatment in patients with $KRAS^{G12C}$-mutant advanced NSCLC. *J Thorac Oncol.* 2024;19(10):S41-S42.
38. Cassier P, et al. KontRASt-01 update: Safety and efficacy of JDQ443 in *KRAS G12C*-mutated solid tumors including non-small cell lung cancer (NSCLC). *Int J Radiat Oncol, Biol Phys.* 2024;118(1):e4-e5.
39. Lorthiois E, et al. JDQ443, a structurally novel, pyrazole-based, covalent inhibitor of KRASG12C for the treatment of solid tumors. *J Med Chem.* 2022;65(24):16173-16203.
40. Cassier PA, et al. KontRASt-01 update: Safety and efficacy of JDQ443 in KRAS G12C-mutated solid tumors including non-small cell lung cancer (NSCLC). *J Clin Oncol.* 2023;41(16_suppl):9007-9007.
41. Liu R, et al. First-in-human study of ZG19018, targeting $KRAS^{G12C}$, as monotherapy in patients with advanced solid tumors. *J Clin Oncol.* 2023;41(16_suppl):e15127-e15127.
42. Zhu B, et al. Abstract 2790: Preclinical studies of ZG19018, a novel irreversible covalent inhibitor of KRAS G12C, for the treatment of advanced non-small cell lung cancer and other solid tumors. *Cancer Res.* 2023;83(7_Supplement):2790-2790.
43. Nagashima T, et al. ASP3082, a first-in-class novel KRAS G12D degrader, exhibits remarkable anti-tumor activity in KRAS G12D mutated cancer models. *Eur J Cancer.* 2022;174:S30.
44. Tolcher AW, et al. Trial in progress: A phase 1, first-in-human, open-label, multicenter, dose-escalation and dose-expansion study of ASP3082 in patients with previously treated advanced solid tumors and KRAS G12D mutations. *J Clin Oncol.* 2023;41(4_suppl):TPS764-TPS764.
45. Nagashima T, et al. Abstract 5735: Novel KRAS G12D degrader ASP3082 demonstrates in vivo, dose-dependent KRAS degradation, KRAS pathway inhibition, and antitumor efficacy in multiple KRAS G12D-mutated cancer models. *Cancer Res.* 2023;83(7_Supplement):5735-5735.
46. Park W, et al. 608O preliminary safety and clinical activity of ASP3082, a first-in-class, KRAS G12D selective protein degrader in adults with advanced pancreatic (PC), colorectal (CRC), and non-small cell lung cancer (NSCLC). *Ann Oncol.* 2024;35:S486-S487.
47. Sen S, et al. Trial in progress: Phase 1 study of the selective protein degrader ASP4396 in patients with locally advanced or metastatic solid tumors with $KRAS^{G12C}$ mutations. *J Clin Oncol.* 2025;43(16_suppl):TPS3178-TPS3178.
48. Martins CP, et al. Abstract ND11: AZD0022: A potent, oral KRASG12D-selective inhibitor that drives robust pathway inhibition and anti-tumour activity in KRASG12D models. *Cancer Res.* 2025;85(8_Supplement_2):ND11-ND11.
49. Yan F, et al. Abstract lb165: GFH547: An orally bioavailable, cyclophilin A-hijacking panRAS(on) inhibitor with broad spectrum anti-tumor activities. *Cancer Res.* 2024;84(7_Supplement):LB165-LB165.
50. Ai X, et al. A first-in-human phase I/II study of GFH375, a highly selective and potent oral KRAS G12D inhibitor in patients with KRAS G12D mutant advanced solid tumors. *J Clin Oncol.* 2025;43(16_suppl):3013-3013.
51. Zhou C, et al. LBA33 a first-in-human phase I study of a novel KRAS G12D inhibitor HRS-4642 in patients with advanced solid tumors harboring KRAS G12D mutation. *Ann Oncol.* 2023;34:S1273.
52. Zhou C, et al. Anti-tumor efficacy of HRS-4642 and its potential combination with proteasome inhibition in KRAS G12D-mutant cancer. *Cancer Cell.* 2024;42(7):1286-1300 e1288.
53. Farren MR, et al. Abstract 5900: INCB161734: A novel, potent, and orally bioavailable KRAS G12D selective inhibitor demonstrates antitumor activity in KRAS G12D mutant tumors. *Cancer Res.* 2024;84(6_Supplement):5900-5900.
54. Iyer C, et al. Abstract B115: Preclinical characterization of LY3962673, an orally bioavailable, highly potent, and selective KRAS G12D inhibitor. *Mol Cancer Ther.* 2023;22(12_Supplement):B115-B115.
55. Gong X, et al. Abstract 3316: LY3962673, an oral, highly potent, mutant-selective, and non-covalent KRAS G12D inhibitor demonstrates robust anti-tumor activity in KRAS G12D models. *Cancer Res.* 2024;84(6_Supplement):3316-3316.
56. Lakhani NJ, et al. MOONRAY-01, a phase 1 study of LY3962673, a potent, orally bioavailable, and selective KRAS G12D inhibitor in KRAS G12D-mutant solid tumors. *J Clin Oncol.* 2025;43(4_suppl):TPS845-TPS845.
57. Hallin J, et al. Anti-tumor efficacy of a potent and selective non-covalent $KRAS^{G12D}$ inhibitor. *Nat Med.* 2022;28(10):2171-2182.
58. Kemp SB, et al. Efficacy of a small-molecule inhibitor of KRASG12D in immunocompetent models of pancreatic cancer. *Cancer Discov.* 2023;13(2):298-311.
59. Wang X, et al. Identification of MRTX1133, a noncovalent, potent, and selective $KRAS^{G12D}$ inhibitor. *J Med Chem.* 2022;65(4):3123-3133.

60. Zhang YJ, et al. Abstract LB_C09: QTX3034, a potent and selective multi-KRAS inhibitor, synergizes with EGFR inhibitors and enhances anti-tumor activity. *Mol Cancer Ther.* 2023;22(12_Supplement):LB_C09-LB_C09.
61. Zhang YW, et al. Abstract lb320: Discovery and characterization of QTX3034, a potent, selective, and orally bioavailable allosteric KRAS inhibitor. *Cancer Res.* 2023;83(8_Supplement):LB320-LB320.
62. Patnaik A, et al. A phase 1 trial evaluating the safety, tolerability, PK, and preliminary efficacy of QTX3034, an oral G12D-preferring multi-KRAS inhibitor, in patients with solid tumors with KRASG12D mutation. *J Clin Oncol.* 2024;42.
63. Vo ED, et al. Abstract lb321: Discovery and characterization of QTX3046, a potent, selective, and orally bioavailable non-covalent KRASG12D inhibitor. *Cancer Res.* 2023;83(8_Supplement):LB321-LB321.
64. Shang E, et al. Abstract 3315: Preclinical studies of TSN1611, a potent, selective, and orally bioavailable KRASG12D inhibitor. *Cancer Res.* 2024;84(6_Supplement):3315-3315.
65. Jiang L, et al. Abstract 526: RMC-9805, a first-in-class, mutant-selective, covalent and oral KRASG12D(ON) inhibitor that induces apoptosis and drives tumor regression in preclinical models of KRASG12D cancers. *Cancer Res.* 2023;83(7_Supplement):526-526.
66. Spira AI, et al. Preliminary safety, antitumor activity, and circulating tumor DNA (ctDNA) changes with RMC-9805, an oral, RAS(ON) G12D-selective tri-complex inhibitor in patients with KRAS G12D pancreatic ductal adenocarcinoma (PDAC) from a phase 1 study in advanced solid tumors. *J Clin Oncol.* 2025;43(4_suppl):724-724.
67. Arbour KC, et al. Abstract CT019: Preliminary safety and antitumor activity of zoldonrasib (RMC-9805), an oral, RAS(ON) G12D-selective, tri-complex inhibitor in patients with KRAS G12D non-small cell lung cancer (NSCLC) from a phase 1 study in advanced solid tumors. *Cancer Res.* 2025;85(8_Supplement_2):CT019-CT019.
68. Zhang YW, et al. Abstract lb163: Discovery and characterization of QTX3544, a potent, selective, and orally bioavailable allosteric G12V preferring multi KRAS inhibitor. *Cancer Res.* 2024;84(7_Supplement):LB163-LB163.
69. Wang T, et al. 8 oral: ALTA3263: An oral, KRAS isoform-selective, dual on/off state, noncovalent inhibitor induces regressions across KRAS G12V, G12D, and G12C cancer models. *Eur J Cancer.* 2024;211:114537.
70. Stahlhut Espinosa CE, et al. Abstract 4378: BBO-11818, an orally bioavailable, highly potent and non-covalent pan-KRAS inhibitor demonstrates robust anti-tumor activity in KRAS-mutant preclinical models. *Cancer Res.* 2025;85(8_Supplement_1):4378-4378.
71. Tedeschi A, et al. Abstract A085: Bi KRASmulti, a first-in-class, orally bioavailable and direct inhibitor of diverse oncogenic KRAS variants drives tumor regression in preclinical models and validates wild-type amplified KRAS as a therapeutic target. *Mol Cancer Ther.* 2023;22(12_Supplement):A085-A085.
72. Gao H, et al. Abstract 4375: LY4066434, an oral small molecule pan-KRAS inhibitor, demonstrates robust anti-tumor activity in KRAS-mutant models, including in the CNS. *Cancer Res.* 2025;85(8_Supplement_1):4375-4375.
73. Jiang J, et al. Translational and therapeutic evaluation of RAS-GTP inhibition by RMC-6236 in RAS-driven cancers. *Cancer Discov.* 2024;14(6):994-1017.
74. Garrido-Laguna I, et al. Safety, efficacy, and on-treatment circulating tumor DNA (ctDNA) changes from a phase 1 study of RMC-6236, a RAS(ON) multi-selective, tri-complex inhibitor, in patients with RAS mutant pancreatic ductal adenocarcinoma (PDAC). *J Clin Oncol.* 2025;43(4_suppl):722-722.
75. Ma Z, et al. RAS(ON) therapies on the horizon to address KRAS resistance: Highlight on a phase III clinical candidate daraxonrasib (RMC-6236). *J Med Chem.* 2025;68(12):12287-12292.
76. Sase H, et al. Abstract 1654: Anti-tumor activity of orally-available cyclic peptide LUNA18 through direct RAS inhibition in RAS-altered tumors. *Cancer Res.* 2024;84(6_Supplement):1654-1654.
77. Xu Z, et al. Abstract 2627: The small molecule KRAS inhibitor, TEB-17231, blocks tumor progression and overcomes KRASG12C inhibitor mediated resistance. *Cancer Res.* 2023;83(7_Supplement):2627-2627.
78. Pant S, et al. First-in-human phase 1 trial of ELI-002 immunotherapy as treatment for subjects with Kirsten rat sarcoma (KRAS)-mutated pancreatic ductal adenocarcinoma and other solid tumors. *J Clin Oncol.* 2022;40(16_suppl):TPS2701-TPS2701.

Supplemental Figure 1

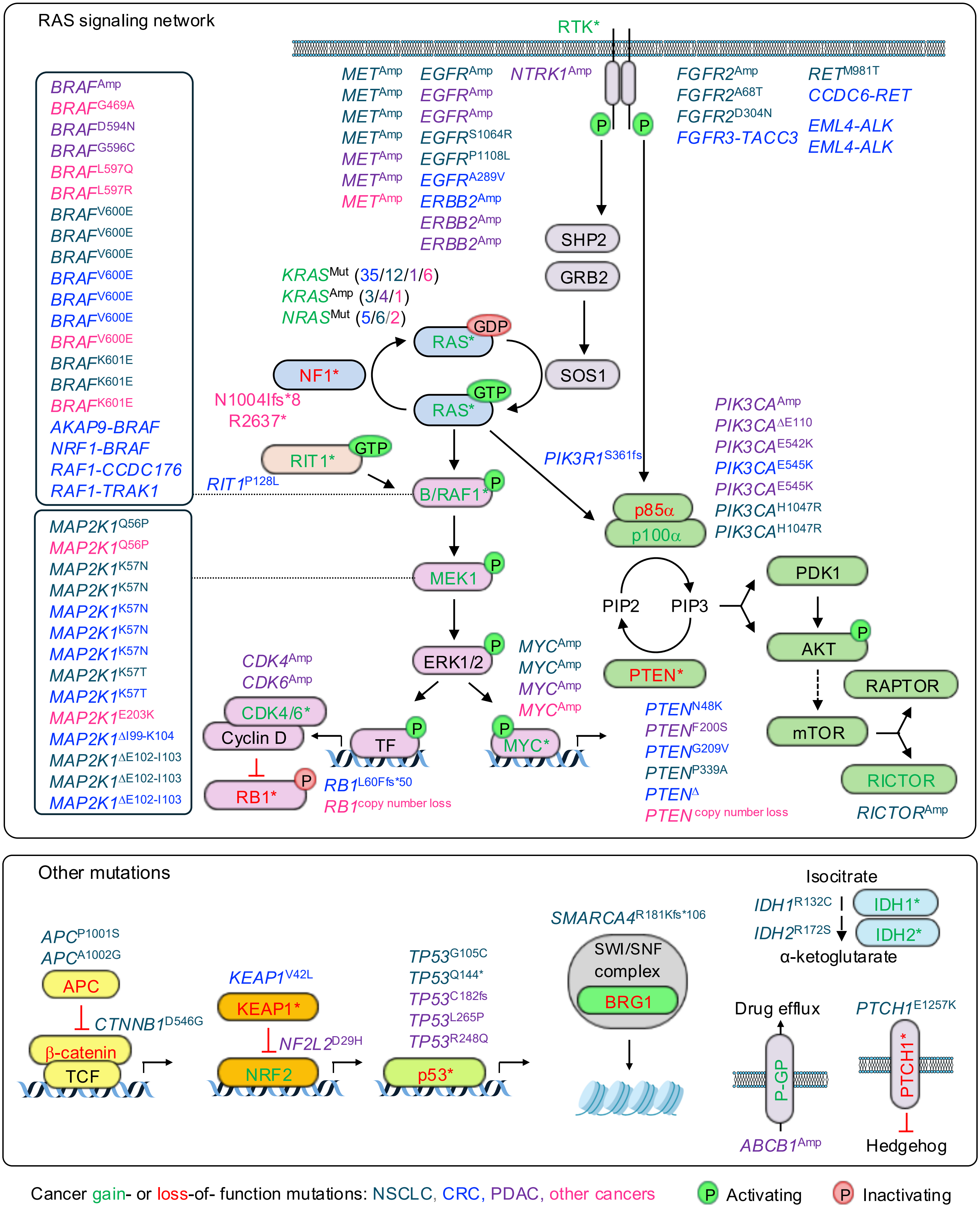

Supplemental Figure 1. Signaling network distribution of all DNA alterations identified in patients that relapsed after treatment with KRAS^{G12C} inhibitors. DNA sequence analyses of biopsy tissue or circulating tumor DNA from 151 patients, who relapsed on treatment with sotorasib, adagrasib, divarasib, or LY3537982, identified the enrichment of 188 total genetic alterations compared with samples from pretreated patients (87, 125, 164, 165, 167, 169). Top panel shows DNA alterations (missense and deletion mutations, gene fusions, amplifications, or copy number loss) according to their position in the RAS signaling network: either upstream of, downstream of, or at the level of RAS itself. Bottom panel shows mutations in other signaling networks. The color coding indicates gain- (green) or loss- (red) of-function of each gene product, as well as the tumor type in which it was identified. Phosphorylation events are also color coded according to whether they are activating (green) or inactivating (red).
